# Correction: The promise of open survey questions—The validation of text-based job satisfaction measures

**DOI:** 10.1371/journal.pone.0236900

**Published:** 2020-07-23

**Authors:** Indy Wijngaards, Martijn Burger, Job van Exel

There is an error in affiliation 1 for authors Indy Wijngaards and Martijn Burger. The correct affiliation is: Erasmus Happiness Economics Research Organization, Erasmus University Rotterdam, Rotterdam, the Netherlands.
