# Supplementary material for: The promise of open survey questions—The validation of text-based job satisfaction measures
Source: PLoS One. 2019 Dec 26;14(12):e0226408. doi: 10.1371/journal.pone.0226408 (PMC6932814; doi:10.1371/journal.pone.0226408)
Supplement: S1 Protocol — (titled [CodingProtocol.pdf]). (DOCX) [file pone.0226408.s001.docx]

**Rating open and semi-open job satisfaction questions**

Besides this instruction, this Excel workbook contains two sheets: one with comments to semi-open questions and one to open questions. For you the task to rate these answers in terms of sentiment. To clarify what I mean with that, I have provided an instruction in the form of a Q&A. Please read them carefully. For questions or remarks, send me an email (…). I am really grateful for your efforts. Thank you.

Q1: What scale will be used to measure the sentiment?

A1: We will both use a 5-point Likert scale with five answer categories: ‘Very negative’, ‘Negative’, ‘Neutral’, ‘Positive’, and ‘Very positive’.

Q2: I see several open columns. What purpose do they serve?

A2: This is where you will write down your ratings. So, for example, first row of Semi1 contains an adjective, first row R1 will contain your rating for that adjective.

Q3: What notation should I use?

A3: Use the numbers that correspond with the categories: 1 = ‘Very negative’, 2 = ‘Negative’, 3 = ‘Neutral’, 4 = ‘Positive’, 5 = ‘Very positive’. Please do not use decimals, please use whole numbers/integers. Using the previous example, adjective in first row of Semi1 will be rated with an integer. An integer in the range of 1 to 5 will be written down in the first row under R1.

Q4: Can I work with other people to share the work?

A4: Most certainly, no. It is very important that you, and you alone, rate all the texts in terms of sentiment. Your friend or family member can have another idea about words. Working together with other person's would heavily bias my results.

Q5: How should I know the difference between e.g. ‘Positive’ and ‘Very positive’?

A5: This will be hard. Sure, things like ‘the best’, ‘magnificent’, ‘fantastic’ suggest a more positive sentiment than ‘good’, ‘fine’ and ‘sufficient’. Still, most of the time you will have to go with your gut. This is perfectly fine, as we are human raters and have our own interpretations of words and word combinations.

Q6: Should I rank the adjectives as context-free words in R1 to R5?

A6: No. The context of the words is very important. The word ‘challenging’ can be combined with ‘stressful’ and ‘hectic’, which means that the word is probably negative or at least neutral. On the other hand, the combination of ‘challenging’, ‘great’ and ‘motivating’ suggests positive sentiment of the word ‘challenging’. Thus, rate the adjectives on sentiment based in the context of the other adjectives.

Q7: Some people used small sentences or did not provide an adjective. What should I do?

A7: Please rate them anyway. They may contain emotion. Words that do not contain any emotion (e.g. 'the') would be best rated neutral. This means that every word should be rated!

Q8: What questions have been asked? And to whom?

A8: The semi-open questions reads "Which three to five adjectives come to mind when you think about your job as a whole?" The open-question reads: "How do you think of your job as a whole?" With respect to the sample size, 997 US workers have filled in the survey. They work at different organization. The survey was anonymous.

Again, thank you a lot!

Name rater: …………

Date finished: ………….
