# Supplementary material for: The promise of open survey questions—The validation of text-based job satisfaction measures
Source: PLoS One. 2019 Dec 26;14(12):e0226408. doi: 10.1371/journal.pone.0226408 (PMC6932814; doi:10.1371/journal.pone.0226408)
Supplement: S1 Dataset — (titled [Data_PromiseOfOpenQuestions.zip]). (ZIP) [file pone.0226408.s003.zip › Data_PromiseOfOpenQuestions/R_markdown_paper.html]

Detecting\_sentiment\_in\_open\_quesitons


# Detecting\_sentiment\_in\_open\_quesitons

#### *Indy Wijngaards*

#### *21 februari 2019*

# Preparation

Loading packages and listing specially designed functions.

```
knitr::opts_chunk$set(echo = TRUE)
options(warn=-1)
```

```
#loading packages
library('dplyr')
library('cowplot')
library('psych')
library('tm')
library('readr')
library('sentimentr')
library('tibble')
library('stringr')
library('scales')
library('tidyr')
library("NLP")
library('openNLP')
library('irr') 
library('openNLPmodels.en')
library('xlsx')

#functions written for analyses
as.numeric.factor <- function(x) {as.numeric(levels(x))[x]}

tagPOS <- function(x, ...) {
    s <- as.String(x)
    word_token_annotator <- Maxent_Word_Token_Annotator()
    a2 <- NLP::Annotation(1L, "sentence", 1L, nchar(s))
    a2 <- NLP::annotate(s, word_token_annotator, a2)
    a3 <- NLP::annotate(s, Maxent_POS_Tag_Annotator(), a2)
    a3w <- a3[a3$type == "word"]
    POStags <- unlist(lapply(a3w$features, `[[`, "POS"))
    return (POStags)
  }

# theme APA
apatheme=theme_bw()+
  theme(panel.grid.major=element_blank(),
        panel.grid.minor=element_blank(),
        panel.border=element_blank(),
        axis.line=element_line(),
        text=element_text(family='serif'),
        legend.title=element_blank())

fn <- function(x) round(rescale(x, to = c(1, 5)),0)
```

# Data loading and preprocessing

## Path

Please adapt the path to your own path

```
path <- "C:/Users/pnl01j1k/Dropbox/Data_PromiseOfOpenQuestions"
```

## Loading data

Two waves of data collection took place.

```
## Wave 1
setwd(paste0(path, "/Wave 1"))
data <- read.csv('Job satisfaction data wave 1.csv', header = TRUE, na.strings=c(" ", ""))

## Wave 2
setwd(paste0(path, "/Wave 2"))
data_w2 <- read.csv('Job satisfaction data wave 2.csv', header = TRUE, na.strings=c(" ", ""))
```

## Preparation data wave 2

The IDs of the respondents in wave 1 had to be collected to collect data for wave 2

```
ID_data <- data
ID_data <- sub("\\@email.prolific.ac*", "",as.character(data$Q31))
ID_data <- ID_data[nchar(as.character(ID_data)) == 24 & as.character(ID_data) != "Nikkimoorish@yahoo.co.uk"]
ID_data <- paste(  paste0(ID_data, ","), collapse = " ")
```

## Filtering, renaming and selecting data

Several variables from the dataset were irrelevant for this study. Therefore they were omitted.

```
data <- dplyr::select(data, c(Q28_1, Q31, Q34, Q33_1, Q33_2,Q33_3, Q33_4, Q33_5, 
             Q7, Q9, Q8, Q12, Q28_2, Q28_3,Q28_4, Q28_5, Q28_6, Q28_7, Q28_8, Q36, Q38, Q39, Q40, Q41, Q42 )) %>%
  slice(13:nrow(data)) %>%
  rename(Job_satisfaction_closed = Q28_1, ProlificID = Q31, Job_satisfaction_open_text = Q34, Job_satisfaction_semi1_text = Q33_1, 
         Job_satisfaction_semi2_text = Q33_2,
         Job_satisfaction_semi3_text = Q33_3, Job_satisfaction_semi4_text = Q33_4, Job_satisfaction_semi5_text = Q33_5,
         
         Education = Q12, Gender = Q8, Marital_status = Q9, Age = Q7, Satisfaction_work_environ = Q28_8, Satisfaction_work_private = Q28_2, 
         Satisfaction_pay = Q28_3, Satisfaction_work_content = Q28_4, Satisfaction_company = Q28_5, Satisfaction_supervisor = Q28_6,
         Satisfaction_team = Q28_7, Resp_open = Q36, Resp_semi1 = Q38, Resp_semi2 = Q39, Resp_semi3 = Q40, Resp_semi4 = Q41, Resp_semi5 = Q42) 

#Wave_2
data_w2 <- dplyr::select(data_w2, c(Approval, Q86, Q88_4, Standalone_open_text, Semi_open_text_1, 
             Semi_open_text_2, Semi_open_text_3,Semi_open_text_4, Semi_open_text_5, Semiopen_rating_1, Semiopen_rating_2, Semiopen_rating_3, Semiopen_rating_4, Semiopen_rating_5, Standalone_rating)) %>%
  slice(7:nrow(data_w2)) %>%
  filter(Approval == 1) %>% 
  rename(ProlificID = Q86, Job_satisfaction_closed = Q88_4, Job_satisfaction_open_text = Standalone_open_text,
         Job_satisfaction_semi1_text = Semi_open_text_1, Job_satisfaction_semi2_text = Semi_open_text_2, 
         Job_satisfaction_semi3_text = Semi_open_text_3, Job_satisfaction_semi4_text = Semi_open_text_4, Job_satisfaction_semi5_text = Semi_open_text_5) %>%
  filter(ProlificID %in% data$ProlificID)
```

## Pre-processing data

### Pasting texts together

The individual respondes to the semi-open questions were merged into a single string and converted into character.

```
data$Job_satisfaction_semi1_5_text <- as.character(with(data, paste0(data$Job_satisfaction_semi1_text, " ",
      data$Job_satisfaction_semi2_text, " ",
      data$Job_satisfaction_semi3_text, " ",
      data$Job_satisfaction_semi4_text, " ",
      data$Job_satisfaction_semi5_text)))
data$Job_satisfaction_semi1_text <- as.character(data$Job_satisfaction_semi1_text)
data$Job_satisfaction_semi2_text <- as.character(data$Job_satisfaction_semi2_text)
data$Job_satisfaction_semi3_text <- as.character(data$Job_satisfaction_semi3_text)
data$Job_satisfaction_semi4_text <- as.character(data$Job_satisfaction_semi5_text)
data$Job_satisfaction_semi5_text <- as.character(data$Job_satisfaction_semi5_text)

data_w2$Job_satisfaction_semi1_5_text <- as.character(with(data_w2, paste0(data_w2$Job_satisfaction_semi1_text, " ",
      data_w2$Job_satisfaction_semi2_text, " ",
      data_w2$Job_satisfaction_semi3_text, " ",
      data_w2$Job_satisfaction_semi4_text, " ",
      data_w2$Job_satisfaction_semi5_text)))
data_w2$Job_satisfaction_semi1_5_text <- gsub("NA", "",data_w2$Job_satisfaction_semi1_5_text)
data_w2$Job_satisfaction_semi1_text <- as.character(data_w2$Job_satisfaction_semi1_text)
data_w2$Job_satisfaction_semi2_text <- as.character(data_w2$Job_satisfaction_semi2_text)
data_w2$Job_satisfaction_semi3_text <- as.character(data_w2$Job_satisfaction_semi3_text)
data_w2$Job_satisfaction_semi4_text <- as.character(data_w2$Job_satisfaction_semi4_text)
data_w2$Job_satisfaction_semi5_text <- as.character(data_w2$Job_satisfaction_semi5_text)
```

### Spelling checks

Respondents made spelling mistakes and typos. To make sure our sentiment analyses were effective, these mistakes were corrected using a manual spelling check in Excel 2016.

```
setwd(paste0(path, "/Wave 1"))

# checked spelling wave 1
data$Job_satisfaction_open_text_LC <- as.vector(read.csv('Spelling_check_open_checked_w1.csv'))$x
data$Job_satisfaction_semi1_5_text_LC <- as.vector(read.csv('Spelling_check_semi_checked_w1.csv'))$x
data$Job_satisfaction_semi1_5_text <- NULL
data$Job_satisfaction_open_text <- NULL

setwd(paste0(path, "/Wave 2"))

# checked spelling wave 2
open <- xlsx::read.xlsx('Spelling_check_open_w2_checked.xlsx', 1, stringsAsFactors=FALSE)
data_w2$Job_satisfaction_open_text_LC <- open$x
data_w2$Job_satisfaction_semi1_5_text_LC <- as.character(as.vector(read.csv('Spelling_check_semi_w2_checked.csv'))$x)
data_w2$Job_satisfaction_semi1_5_text <- NULL
data_w2$Job_satisfaction_open_text <- NULL
```

### Cleaning texts

We converted all strings into lowercase and made sure we only used alphabetical characters.

```
### WAVE 1 ###

# semi-open questions:joining the words, deleting interpunction, all to lower case
data$Job_satisfaction_semi1_5_text_LC <- gsub("\\s+", " ", data$Job_satisfaction_semi1_5_text_LC)
data$Job_satisfaction_semi1_5_text_LC <- str_replace_all(data$Job_satisfaction_semi1_5_text_LC, "[^[:alnum:]]", " ")
data$Job_satisfaction_semi1_5_text_LC <- str_replace_all(data$Job_satisfaction_semi1_5_text_LC, "[\r\n]" , "")

data[c('Job_satisfaction_semi1_text','Job_satisfaction_semi2_text',
       'Job_satisfaction_semi3_text', 'Job_satisfaction_semi4_text', 
       'Job_satisfaction_semi5_text')] <- as.data.frame(lapply(X = data[c('Job_satisfaction_semi1_text',
      'Job_satisfaction_semi2_text',
      'Job_satisfaction_semi3_text', 
      'Job_satisfaction_semi4_text', 
      'Job_satisfaction_semi5_text')], FUN = 
 function(y) gsub("\\s+", " ", y)))
data[c('Job_satisfaction_semi1_text','Job_satisfaction_semi2_text',
       'Job_satisfaction_semi3_text', 'Job_satisfaction_semi4_text', 
       'Job_satisfaction_semi5_text')] <- as.data.frame(lapply(X = data[c('Job_satisfaction_semi1_text',
      'Job_satisfaction_semi2_text',
      'Job_satisfaction_semi3_text', 
      'Job_satisfaction_semi4_text', 
      'Job_satisfaction_semi5_text')], FUN = 
 function(y) gsub("[^[:alnum:]]", " ", y)))
data[c('Job_satisfaction_semi1_text','Job_satisfaction_semi2_text',
       'Job_satisfaction_semi3_text', 'Job_satisfaction_semi4_text', 
       'Job_satisfaction_semi5_text')] <- as.data.frame(lapply(X = data[c('Job_satisfaction_semi1_text',
      'Job_satisfaction_semi2_text',
      'Job_satisfaction_semi3_text', 
      'Job_satisfaction_semi4_text', 
      'Job_satisfaction_semi5_text')], FUN = 
 function(y) gsub("[\r\n]", " ", y)))

data[c('Job_satisfaction_semi1_text','Job_satisfaction_semi2_text',
       'Job_satisfaction_semi3_text', 'Job_satisfaction_semi4_text', 
       'Job_satisfaction_semi5_text')] <- lapply(X = data[c('Job_satisfaction_semi1_text',
      'Job_satisfaction_semi2_text',
      'Job_satisfaction_semi3_text', 
      'Job_satisfaction_semi4_text', 
      'Job_satisfaction_semi5_text')], as.character)


#open question: deleting interpunction, all to lower case
data$Job_satisfaction_open_text_LC <- gsub("n't", " not", data$Job_satisfaction_open_text_LC, fixed = TRUE)
data$Job_satisfaction_open_text_LC <- tolower(gsub("5980e543fdb524000114fcd1", " ", data$Job_satisfaction_open_text_LC, fixed = TRUE))
data$Job_satisfaction_open_text_LC <- gsub("\\s+", " ", data$Job_satisfaction_open_text_LC)
data$Job_satisfaction_open_text_LC <- str_replace_all(data$Job_satisfaction_open_text_LC, "[^[:alnum:]]", " ")
data$Job_satisfaction_open_text_LC <- str_replace_all(data$Job_satisfaction_open_text_LC, "[\r\n]" , "")

### WAVE 2 ###

# semi-open questions:joining the words, deleting interpunction, all to lower case
data_w2$Job_satisfaction_semi1_5_text_LC <- gsub("\\s+", " ", data_w2$Job_satisfaction_semi1_5_text_LC)
data_w2$Job_satisfaction_semi1_5_text_LC <- str_replace_all(data_w2$Job_satisfaction_semi1_5_text_LC, "[^[:alnum:]]", " ")
data_w2$Job_satisfaction_semi1_5_text_LC <- tolower(str_replace_all(data_w2$Job_satisfaction_semi1_5_text_LC, "[\r\n]" , ""))

#open question: deleting interpunction, all to lower case
data_w2$Job_satisfaction_open_text_LC <- gsub("n't", " not", data_w2$Job_satisfaction_open_text_LC, fixed = TRUE)
data_w2$Job_satisfaction_open_text_LC <- str_replace_all(tolower(data_w2$Job_satisfaction_open_text_LC), "[^[:alnum:]]", " ")
data_w2$Job_satisfaction_open_text_LC <- str_replace_all(data_w2$Job_satisfaction_open_text_LC, "[\r\n]" , "")
```

# Exploring the texts

As an illustration of the textual data, we examined the mean and median number of words used and calculated the corresponding standard deviation. In addition, we used part-of-speech (POS) tagging to find out whether respondents wrote down adjectives to the semi-open question.

## Semi-open

```
#Create vectors with all texts
semi_open_question_text_w1 <- as.character(data$Job_satisfaction_semi1_5_text_LC)
semi_open_question_text_w2 <- as.character(data_w2$Job_satisfaction_semi1_5_text_LC)

#Median, mean and standerd deviation: Wave 1
median_SO_w1 <- round(median(sapply(strsplit(semi_open_question_text_w1, " "), length)),2)
median_SO_w1
## [1] 5
mean_SO_w1 <- round(mean(sapply(strsplit(semi_open_question_text_w1, " "), length)),2)
mean_SO_w1
## [1] 4.65
sd_SO_w1 <- round(sd(sapply(strsplit(semi_open_question_text_w1, " "), length)), 2)
sd_SO_w1
## [1] 1.93

#Median, mean and standerd deviation: Wave 2
median_SO_w2 <- round(median(sapply(strsplit(semi_open_question_text_w2, " "), length)),2)
median_SO_w2
## [1] 5
mean_SO_w2 <- round(mean(sapply(strsplit(semi_open_question_text_w2, " "), length)),2)
mean_SO_w2
## [1] 4.45
sd_SO_w2 <- round(sd(sapply(strsplit(semi_open_question_text_w2, " "), length)), 2)
sd_SO_w2
## [1] 1.62

#Table of word categories: wave 1
SO_string_w1 = paste(semi_open_question_text_w1, collapse=" ")

tagged_str <- tagPOS(SO_string_w1)
freq_table_SO <- as.data.frame(ftable(tagged_str))
freq_table_SO$pct <- round(x = (freq_table_SO$Freq/sum(freq_table_SO$Freq))*100, digits = 2)
freq_table_SO
##    tagged_str Freq   pct
## 1           .    1  0.02
## 2          CC    4  0.09
## 3          CD    3  0.06
## 4          DT   34  0.73
## 5          IN   67  1.45
## 6          JJ 2588 55.88
## 7         JJR    3  0.06
## 8         JJS    3  0.06
## 9          MD    5  0.11
## 10         NN  908 19.61
## 11        NNS   91  1.97
## 12        PRP   18  0.39
## 13       PRP$   12  0.26
## 14         RB  114  2.46
## 15        RBR    4  0.09
## 16         RP    4  0.09
## 17         TO   17  0.37
## 18         VB   33  0.71
## 19        VBD   63  1.36
## 20        VBG  473 10.21
## 21        VBN  139  3.00
## 22        VBP   31  0.67
## 23        VBZ   13  0.28
## 24        WRB    3  0.06

#Table of word categories: wave 2
SO_string_w2 = paste(semi_open_question_text_w2, collapse=" ")

tagged_str <- tagPOS(SO_string_w2)
freq_table_SO <- as.data.frame(ftable(tagged_str))
freq_table_SO$pct <- round(x = (freq_table_SO$Freq/sum(freq_table_SO$Freq))*100, digits = 2)
freq_table_SO
##    tagged_str Freq   pct
## 1          CC    1  0.19
## 2          DT    5  0.97
## 3          IN    5  0.97
## 4          JJ  310 60.08
## 5          NN   92 17.83
## 6         NNS    5  0.97
## 7          RB   16  3.10
## 8          VB    1  0.19
## 9         VBD    6  1.16
## 10        VBG   53 10.27
## 11        VBN   19  3.68
## 12        VBP    3  0.58
```

## Open

```
open_question_text_w1 <- as.character(data$Job_satisfaction_open_text_LC)

#Median, mean and standerd deviation: Wave 1
median_O_w1 <- round(median(sapply(strsplit(open_question_text_w1, " "), length)),2)
median_O_w1
## [1] 39
mean_O_w1 <- round(mean(sapply(strsplit(open_question_text_w1, " "), length)),2)
mean_O_w1
## [1] 48.47
sd_O_w1 <- round(sd(sapply(strsplit(open_question_text_w1, " "), length), na.rm = TRUE), 2)
sd_O_w1
## [1] 39.72
open_question_text_w2 <- as.character(data_w2$Job_satisfaction_open_text_LC)

#Median, mean and standerd deviation: Wave 2
median_O_w2 <- round(median(sapply(strsplit(open_question_text_w2, " "), length)),2)
median_O_w2
## [1] 57
mean_O_w2 <- round(mean(sapply(strsplit(open_question_text_w2, " "), length)),2)
mean_O_w2
## [1] 64.99
sd_O_w2 <- round(sd(sapply(strsplit(open_question_text_w2, " "), length), na.rm = TRUE), 2)
sd_O_w2
## [1] 42.12
```

# Sentiment analyses

We used four types of sentiment analysis: Independent manual coding, SentimentR, SentiStrength and LIWC.

## Independent coders’ ratings

Three independent raters (Sasqia, Ivonne and Miranda) independently coded all responses. These ratings were averaged and rounded to the nearest number.

### Wave 1

```
setwd(paste0(path, "/Wave 1"))
Raters_semi_open <- as.data.frame(read.csv(file = 'Miranda & Sasqia & Ivonne Semi Open Ratings_w1.csv', sep = ";", na.strings = NA, stringsAsFactors = FALSE))[1:997,]
Raters_open <- as.data.frame(read.csv(file = 'Miranda Sasqia & Ivonne Open_w1.csv', sep = ";", na.strings = NA, stringsAsFactors = FALSE))

data <- bind_cols(data, Raters_semi_open[, c(2:4,6:8,10:12,14:16, 18:23)])
data <- bind_cols(data, Raters_open[, c(2:4)])
data$Rating_Sasqia_semi1 <- as.numeric(gsub(54, 5, data$Rating_Sasqia_semi1))
```

```
data$Job_satisfaction_semi1_overall_raters <- data$Job_satisfaction_semiopen1_5_rater1 <- rowMeans(data[,c("Rating_Miranda_semi1", "Rating_Sasqia_semi1", "Rating_Ivonne_semi1")], na.rm = TRUE)

data$Job_satisfaction_semi2_overall_raters <- data$Job_satisfaction_semiopen1_5_rater1 <- rowMeans(data[,c("Rating_Miranda_semi2", "Rating_Sasqia_semi2", "Rating_Ivonne_semi2")], na.rm = TRUE)

data$Job_satisfaction_semi3_overall_raters <- data$Job_satisfaction_semiopen1_5_rater1 <- rowMeans(data[,c("Rating_Miranda_semi3", "Rating_Sasqia_semi3", "Rating_Ivonne_semi3")], na.rm = TRUE)

data$Job_satisfaction_semi4_overall_raters <- data$Job_satisfaction_semiopen1_5_rater1 <- rowMeans(data[,c("Rating_Miranda_semi4", "Rating_Sasqia_semi4", "Rating_Ivonne_semi4")], na.rm = TRUE)

data$Job_satisfaction_semi5_overall_raters <- data$Job_satisfaction_semiopen1_5_rater1 <- rowMeans(data[,c("Rating_Miranda_semi5", "Rating_Sasqia_semi5", "Rating_Ivonne_semi5")], na.rm = TRUE)


data$Job_satisfaction_semiopen1_5_rater1 <- rowMeans(data[,c("Rating_Miranda_semi1", "Rating_Miranda_semi2", "Rating_Miranda_semi3", "Rating_Miranda_semi4", "Rating_Miranda_semi5")], na.rm = TRUE)

data$Job_satisfaction_semiopen1_5_rater2 <- rowMeans(data[,c("Rating_Sasqia_semi1", "Rating_Sasqia_semi2", "Rating_Sasqia_semi3", "Rating_Sasqia_semi4", "Rating_Sasqia_semi5")],na.rm = TRUE)

data$Job_satisfaction_semiopen1_5_rater3 <- rowMeans(data[,c("Rating_Ivonne_semi1", "Rating_Ivonne_semi2","Rating_Ivonne_semi3", "Rating_Ivonne_semi4", "Rating_Ivonne_semi5")], na.rm = TRUE)

data$Job_satisfaction_semiopen1_5_raters <- round(rowMeans(data[,c("Job_satisfaction_semiopen1_5_rater1",       "Job_satisfaction_semiopen1_5_rater2", "Job_satisfaction_semiopen1_5_rater3")], na.rm = TRUE))


data$Job_satisfaction_open_raters <- round(rowMeans(data[,c('Rating_Ivonne_open', 'Rating_Sasqia_open', 'Rating_Miranda_open')], na.rm = TRUE))

#mean and standard deviation: open
mean(round(data$Job_satisfaction_open_raters))
## [1] 3.352056
sd(round(data$Job_satisfaction_open_raters))
## [1] 1.090958

#mean and standard deviation: semi-open
mean(round(data$Job_satisfaction_semiopen1_5_raters))
## [1] 3.160481
sd(round(data$Job_satisfaction_semiopen1_5_raters))
## [1] 0.9108466
psych::alpha(data[,c('Job_satisfaction_semi1_overall_raters','Job_satisfaction_semi2_overall_raters', 'Job_satisfaction_semi3_overall_raters', 'Job_satisfaction_semi4_overall_raters', 'Job_satisfaction_semi5_overall_raters')])$total$raw_alpha
## [1] 0.9198019

#Inter rater reliability, i.e., ICC - Semi-open
semi1ICC <- as.data.frame(data[c('Rating_Miranda_semi1', 'Rating_Sasqia_semi1', 'Rating_Ivonne_semi1')])
semi2ICC <- as.data.frame(data[c('Rating_Miranda_semi2', 'Rating_Sasqia_semi2', 'Rating_Ivonne_semi2')])
semi3ICC <- as.data.frame(data[c('Rating_Miranda_semi3', 'Rating_Sasqia_semi3', 'Rating_Ivonne_semi3')])
semi4ICC <- as.data.frame(data[c('Rating_Miranda_semi4', 'Rating_Sasqia_semi4', 'Rating_Ivonne_semi4')])
semi5ICC <- as.data.frame(data[c('Rating_Miranda_semi5', 'Rating_Sasqia_semi5', 'Rating_Ivonne_semi5')])
semiallICC <- as.data.frame(data[c('Job_satisfaction_semiopen1_5_rater1', 'Job_satisfaction_semiopen1_5_rater2', 'Job_satisfaction_semiopen1_5_rater3')])
openICC <- as.data.frame(data[c('Rating_Miranda_open', 'Rating_Sasqia_open', 'Rating_Ivonne_open')])

#Inter rater reliability, i.e., ICC - Open
irr::icc(semi1ICC, model = 'twoway', type = 'consistency', unit = 'average')
##  Average Score Intraclass Correlation
## 
##    Model: twoway 
##    Type : consistency 
## 
##    Subjects = 994 
##      Raters = 3 
##    ICC(C,3) = 0.909
## 
##  F-Test, H0: r0 = 0 ; H1: r0 > 0 
## F(993,1986) = 11 , p = 0 
## 
##  95%-Confidence Interval for ICC Population Values:
##   0.899 < ICC < 0.919
irr::icc(semi2ICC, model = 'twoway', type = 'consistency', unit = 'average')
##  Average Score Intraclass Correlation
## 
##    Model: twoway 
##    Type : consistency 
## 
##    Subjects = 995 
##      Raters = 3 
##    ICC(C,3) = 0.902
## 
##  F-Test, H0: r0 = 0 ; H1: r0 > 0 
## F(994,1988) = 10.2 , p = 0 
## 
##  95%-Confidence Interval for ICC Population Values:
##   0.891 < ICC < 0.912
irr::icc(semi3ICC, model = 'twoway', type = 'consistency', unit = 'average')
##  Average Score Intraclass Correlation
## 
##    Model: twoway 
##    Type : consistency 
## 
##    Subjects = 993 
##      Raters = 3 
##    ICC(C,3) = 0.904
## 
##  F-Test, H0: r0 = 0 ; H1: r0 > 0 
## F(992,1984) = 10.4 , p = 0 
## 
##  95%-Confidence Interval for ICC Population Values:
##   0.893 < ICC < 0.914
irr::icc(semi4ICC, model = 'twoway', type = 'consistency', unit = 'average')
##  Average Score Intraclass Correlation
## 
##    Model: twoway 
##    Type : consistency 
## 
##    Subjects = 511 
##      Raters = 3 
##    ICC(C,3) = 0.817
## 
##  F-Test, H0: r0 = 0 ; H1: r0 > 0 
## F(510,1020) = 5.46 , p = 3.82e-117 
## 
##  95%-Confidence Interval for ICC Population Values:
##   0.787 < ICC < 0.843
irr::icc(semi5ICC, model = 'twoway', type = 'consistency', unit = 'average')
##  Average Score Intraclass Correlation
## 
##    Model: twoway 
##    Type : consistency 
## 
##    Subjects = 510 
##      Raters = 3 
##    ICC(C,3) = 0.903
## 
##  F-Test, H0: r0 = 0 ; H1: r0 > 0 
## F(509,1018) = 10.3 , p = 1.62e-212 
## 
##  95%-Confidence Interval for ICC Population Values:
##   0.887 < ICC < 0.917
irr::icc(semiallICC, model = 'twoway', type = 'consistency', unit = 'average')
##  Average Score Intraclass Correlation
## 
##    Model: twoway 
##    Type : consistency 
## 
##    Subjects = 995 
##      Raters = 3 
##    ICC(C,3) = 0.934
## 
##  F-Test, H0: r0 = 0 ; H1: r0 > 0 
## F(994,1988) = 15.2 , p = 0 
## 
##  95%-Confidence Interval for ICC Population Values:
##   0.927 < ICC < 0.941
irr::icc(openICC, model = 'twoway', type = 'consistency', unit = 'average')
##  Average Score Intraclass Correlation
## 
##    Model: twoway 
##    Type : consistency 
## 
##    Subjects = 996 
##      Raters = 3 
##    ICC(C,3) = 0.921
## 
##  F-Test, H0: r0 = 0 ; H1: r0 > 0 
## F(995,1990) = 12.6 , p = 0 
## 
##  95%-Confidence Interval for ICC Population Values:
##   0.912 < ICC < 0.929
```

### Wave 2

```
setwd(paste0(path, "/Wave 2"))

Raters_semi_open <- as.data.frame(read.xlsx(file = 'Wave_2_manual_coding.xlsx', sheetIndex =  2, na.strings = NA, stringsAsFactors = FALSE))[1:116,]
Raters_open <- as.data.frame(read.xlsx(file = 'Wave_2_manual_coding.xlsx', sheetIndex =  3, na.strings = NA, stringsAsFactors = FALSE))[1:116,]

data_w2 <- bind_cols(data_w2, Raters_semi_open[, c(2:4,6:8,10:12,14:16, 18:20)])
data_w2 <- bind_cols(data_w2, Raters_open[, c(2:4)])
```

```
data_w2$Job_satisfaction_semi1_overall_raters <- data_w2$Job_satisfaction_semiopen1_5_rater1 <- rowMeans(data_w2[,c("R1_Ivonne", "R1_Miranda", "R1_Sasqia")], na.rm = TRUE)

data_w2$Job_satisfaction_semi2_overall_raters <- data_w2$Job_satisfaction_semiopen1_5_rater1 <- rowMeans(data_w2[,c("R2_Ivonne", "R2_Miranda", "R1_Sasqia")], na.rm = TRUE)

data_w2$Job_satisfaction_semi3_overall_raters <- data_w2$Job_satisfaction_semiopen1_5_rater1 <- rowMeans(data_w2[,c("R3_Ivonne", "R3_Miranda", "R1_Sasqia")], na.rm = TRUE)

data_w2$Job_satisfaction_semi4_overall_raters <- data_w2$Job_satisfaction_semiopen1_5_rater1 <- rowMeans(data_w2[,c("R4_Ivonne", "R4_Miranda", "R1_Sasqia")], na.rm = TRUE)

data_w2$Job_satisfaction_semi5_overall_raters <- data_w2$Job_satisfaction_semiopen1_5_rater1 <- rowMeans(data_w2[,c("R5_Ivonne", "R5_Miranda", "R1_Sasqia")], na.rm = TRUE)


data_w2$Job_satisfaction_semiopen1_5_rater1 <- rowMeans(data_w2[,c("R1_Ivonne", "R2_Ivonne", "R3_Ivonne", "R4_Ivonne", 'R5_Ivonne')], na.rm = TRUE)
data_w2$Job_satisfaction_semiopen1_5_rater2 <- rowMeans(data_w2[,c("R1_Miranda", "R2_Miranda","R3_Miranda","R4_Miranda","R5_Miranda")], na.rm = TRUE)
data_w2$Job_satisfaction_semiopen1_5_rater3 <- rowMeans(data_w2[,c("R1_Sasqia","R2_Sasqia","R3_Sasqia","R4_Sasqia","R5_Sasqia")], na.rm = TRUE)
data_w2$Job_satisfaction_semiopen1_5_raters <- round(rowMeans(data_w2[,c("Job_satisfaction_semiopen1_5_rater1", "Job_satisfaction_semiopen1_5_rater2", "Job_satisfaction_semiopen1_5_rater3")], na.rm = TRUE))
data_w2$Job_satisfaction_open_raters <- round(rowMeans(data_w2[,c('R_Ivonne', 'R_Sasqia', 'R_Miranda')], na.rm = TRUE))

#some descrptives: open
mean(data_w2$Job_satisfaction_open_raters)
## [1] 3.241379
sd(round(data_w2$Job_satisfaction_open_raters))
## [1] 1.302978

#some descrptives: semi-open
mean(data_w2$Job_satisfaction_semiopen1_5_raters)
## [1] 3.008621
sd(data_w2$Job_satisfaction_semiopen1_5_raters)
## [1] 0.9277902
psych::alpha(data_w2[,c('Job_satisfaction_semi1_overall_raters','Job_satisfaction_semi2_overall_raters', 'Job_satisfaction_semi3_overall_raters', 'Job_satisfaction_semi4_overall_raters', 'Job_satisfaction_semi5_overall_raters')])$total$raw_alpha
## [1] 0.952513

#IRR
semi1ICC <- as.data.frame(data_w2[c("R1_Ivonne", "R1_Miranda", "R1_Sasqia")])
semi2ICC <- as.data.frame(data_w2[c("R2_Ivonne", "R2_Miranda", "R2_Sasqia")])
semi3ICC <- as.data.frame(data_w2[c("R3_Ivonne", "R3_Miranda", "R3_Sasqia")])
semi4ICC <- as.data.frame(data_w2[c("R4_Ivonne", "R4_Miranda", "R4_Sasqia")])
semi5ICC <- as.data.frame(data_w2[c("R5_Ivonne", "R5_Miranda", "R5_Sasqia")])
semiallICC <- as.data.frame(data_w2[c('Job_satisfaction_semiopen1_5_rater1', 'Job_satisfaction_semiopen1_5_rater2', 'Job_satisfaction_semiopen1_5_rater3')])
openICC <- as.data.frame(data_w2[c('R_Ivonne', 'R_Sasqia', 'R_Miranda')])

#icc
irr::icc(semi1ICC, model = 'twoway', type = 'consistency', unit = 'average')
##  Average Score Intraclass Correlation
## 
##    Model: twoway 
##    Type : consistency 
## 
##    Subjects = 116 
##      Raters = 3 
##    ICC(C,3) = 0.918
## 
##  F-Test, H0: r0 = 0 ; H1: r0 > 0 
##  F(115,230) = 12.3 , p = 1.78e-56 
## 
##  95%-Confidence Interval for ICC Population Values:
##   0.889 < ICC < 0.941
irr::icc(semi2ICC, model = 'twoway', type = 'consistency', unit = 'average')
##  Average Score Intraclass Correlation
## 
##    Model: twoway 
##    Type : consistency 
## 
##    Subjects = 115 
##      Raters = 3 
##    ICC(C,3) = 0.914
## 
##  F-Test, H0: r0 = 0 ; H1: r0 > 0 
##  F(114,228) = 11.7 , p = 4.72e-54 
## 
##  95%-Confidence Interval for ICC Population Values:
##   0.883 < ICC < 0.938
irr::icc(semi3ICC, model = 'twoway', type = 'consistency', unit = 'average')
##  Average Score Intraclass Correlation
## 
##    Model: twoway 
##    Type : consistency 
## 
##    Subjects = 115 
##      Raters = 3 
##    ICC(C,3) = 0.935
## 
##  F-Test, H0: r0 = 0 ; H1: r0 > 0 
##  F(114,228) = 15.4 , p = 3.98e-65 
## 
##  95%-Confidence Interval for ICC Population Values:
##   0.911 < ICC < 0.953
irr::icc(semi4ICC, model = 'twoway', type = 'consistency', unit = 'average')
##  Average Score Intraclass Correlation
## 
##    Model: twoway 
##    Type : consistency 
## 
##    Subjects = 86 
##      Raters = 3 
##    ICC(C,3) = 0.91
## 
##  F-Test, H0: r0 = 0 ; H1: r0 > 0 
##   F(85,170) = 11.1 , p = 1.8e-39 
## 
##  95%-Confidence Interval for ICC Population Values:
##   0.871 < ICC < 0.939
irr::icc(semi5ICC, model = 'twoway', type = 'consistency', unit = 'average')
##  Average Score Intraclass Correlation
## 
##    Model: twoway 
##    Type : consistency 
## 
##    Subjects = 71 
##      Raters = 3 
##    ICC(C,3) = 0.917
## 
##  F-Test, H0: r0 = 0 ; H1: r0 > 0 
##   F(70,140) = 12.1 , p = 8.02e-35 
## 
##  95%-Confidence Interval for ICC Population Values:
##   0.877 < ICC < 0.946
irr::icc(semiallICC, model = 'twoway', type = 'consistency', unit = 'average')
##  Average Score Intraclass Correlation
## 
##    Model: twoway 
##    Type : consistency 
## 
##    Subjects = 116 
##      Raters = 3 
##    ICC(C,3) = 0.936
## 
##  F-Test, H0: r0 = 0 ; H1: r0 > 0 
##  F(115,230) = 15.7 , p = 2.04e-66 
## 
##  95%-Confidence Interval for ICC Population Values:
##   0.913 < ICC < 0.954
irr::icc(openICC, model = 'twoway', type = 'consistency', unit = 'average')
##  Average Score Intraclass Correlation
## 
##    Model: twoway 
##    Type : consistency 
## 
##    Subjects = 116 
##      Raters = 3 
##    ICC(C,3) = 0.951
## 
##  F-Test, H0: r0 = 0 ; H1: r0 > 0 
##  F(115,230) = 20.5 , p = 6.88e-78 
## 
##  95%-Confidence Interval for ICC Population Values:
##   0.934 < ICC < 0.965
```

## LIWC

The Linguistic Inquiry and Word Count software was used to compute sentiment scores.

### Wave 1

```
setwd(paste0(path, "/LIWC_results"))

#creating one large dataframe with all results per row
LIWC_data <- list.files(full.names = TRUE, pattern = c("study_1|study1_checked")) %>% 
  lapply(read.csv, na.strings = c("No comment", "no comment", "", "NA")) %>% 
  bind_cols 

LIWC_data <- LIWC_data[, grep("Tone", names(LIWC_data), value = TRUE)]
LIWC_data <- rename(LIWC_data, Job_satisfaction_semiopen1_5_LIWC = Tone, Job_satisfaction_semiopen1_LIWC = Tone1,
    Job_satisfaction_semiopen2_LIWC = Tone2, Job_satisfaction_semiopen3_LIWC = Tone3, 
    Job_satisfaction_semiopen4_LIWC = Tone4, Job_satisfaction_semiopen5_LIWC = Tone5, 
    Job_satisfaction_open_LIWC = Tone6)
LIWC_data <- lapply(LIWC_data, function(y) gsub(",", ".",y)) %>%
  lapply(as.numeric) %>%
  lapply(fn)
  
#merge data into existing dataset
data <- bind_cols(data, LIWC_data)

data<- mutate(data, Job_satisfaction_semiopen4_LIWC = ifelse(is.na(Resp_semi4), NA,
            Job_satisfaction_semiopen4_LIWC),
         Job_satisfaction_semiopen_LIWC = ifelse(is.na(Resp_semi5), NA,
            Job_satisfaction_semiopen5_LIWC))
         
# descrptives open
mean(data$Job_satisfaction_open_LIWC)
## [1] 4.210632
sd(data$Job_satisfaction_open_LIWC)
## [1] 1.339007

# descrptives semi-open
mean(data$Job_satisfaction_semiopen1_5_LIWC)
## [1] 3.693079
sd(data$Job_satisfaction_semiopen1_5_LIWC)
## [1] 1.732654
psych::alpha(data[,c('Job_satisfaction_semiopen1_LIWC','Job_satisfaction_semiopen2_LIWC', 'Job_satisfaction_semiopen3_LIWC', 'Job_satisfaction_semiopen4_LIWC', 'Job_satisfaction_semiopen5_LIWC')])$total$raw_alpha
## [1] 0.4941536
```

### Wave 2

```
setwd(paste0(path, "/LIWC_results"))

#creating one large dataframe with all results per row
LIWC_data <- list.files(full.names = TRUE, pattern = c("w2")) %>% 
  lapply(read.csv, na.strings = c("No comment", "no comment", "", "NA")) %>% 
  bind_cols 

LIWC_data <- LIWC_data[, grep("Tone", names(LIWC_data), value = TRUE)]

LIWC_data <- rename(LIWC_data, Job_satisfaction_semiopen1_5_LIWC_w2 = Tone, Job_satisfaction_semiopen1_LIWC_w2 =
    Tone1,Job_satisfaction_semiopen2_LIWC_w2 = Tone2, Job_satisfaction_semiopen3_LIWC_w2 = Tone3, 
    Job_satisfaction_semiopen4_LIWC_w2 = Tone4, Job_satisfaction_semiopen5_LIWC_w2 = Tone5, 
    Job_satisfaction_open_LIWC_w2 = Tone6) %>%
  slice(2:n())

LIWC_data <- lapply(LIWC_data, function(y) gsub(",", ".",y)) %>%
  lapply(as.numeric) %>%
  lapply(fn)
  

#merge data into existing dataset
data_w2 <- bind_cols(data_w2, LIWC_data)

data_w2 <- 
  mutate(data_w2, Job_satisfaction_semiopen4_LIWC_w2 = ifelse(is.na(Semiopen_rating_4), NA,
            Job_satisfaction_semiopen4_LIWC_w2),
         Job_satisfaction_semiopen5_LIWC_w2 = ifelse(is.na(Semiopen_rating_5), NA,
            Job_satisfaction_semiopen5_LIWC_w2))
         

#mean and standard deviation: open
mean(data_w2$Job_satisfaction_open_LIWC)
## [1] 3.767241
sd(data_w2$Job_satisfaction_open_LIWC)
## [1] 1.5512

#mean and standard deviation: semi-open
mean(data_w2$Job_satisfaction_semiopen1_5_LIWC)
## [1] 3.163793
sd(data_w2$Job_satisfaction_semiopen1_5_LIWC)
## [1] 1.874051
psych::alpha(data_w2[,c('Job_satisfaction_semiopen1_LIWC_w2','Job_satisfaction_semiopen2_LIWC_w2', 'Job_satisfaction_semiopen3_LIWC_w2', 'Job_satisfaction_semiopen4_LIWC_w2', 'Job_satisfaction_semiopen5_LIWC_w2')])$total$raw_alpha
## [1] 0.6880826
```

## Sentimentr

### Wave 1

```
#semi 
data$Job_satisfaction_semiopen1_5_sentimentr <- fn(sentimentr::sentiment(text.var = as.character(data$Job_satisfaction_semi1_5_text_LC))$sentiment)
data$Job_satisfaction_semiopen1_sentimentr <- fn(sentimentr::sentiment(text.var = as.character(data$Job_satisfaction_semi1_text))$sentiment)
data$Job_satisfaction_semiopen2_sentimentr <- fn(sentimentr::sentiment(text.var = as.character(data$Job_satisfaction_semi2_text))$sentiment)
data$Job_satisfaction_semiopen3_sentimentr <- fn(sentimentr::sentiment(text.var = as.character(data$Job_satisfaction_semi3_text))$sentiment)
data$Job_satisfaction_semiopen4_sentimentr <- fn(sentimentr::sentiment(text.var = as.character(data$Job_satisfaction_semi4_text))$sentiment)
data$Job_satisfaction_semiopen5_sentimentr <- fn(sentimentr::sentiment(text.var = as.character(data$Job_satisfaction_semi5_text))$sentiment)

#open
data$Job_satisfaction_open_sentimentr <- fn(sentimentr::sentiment(text.var = data$Job_satisfaction_open_text_LC)$sentiment)

data <- mutate(data, Job_satisfaction_semiopen4_sentimentr = ifelse(is.na(Resp_semi4), NA, Job_satisfaction_semiopen4_sentimentr),Job_satisfaction_semiopen5_sentimentr = ifelse(is.na(Resp_semi4), NA, Job_satisfaction_semiopen5_sentimentr))
         
#mean and standard deviation: open
mean(data$Job_satisfaction_open_sentimentr)
## [1] 2.810431
sd(data$Job_satisfaction_open_sentimentr)
## [1] 0.6170043

#mean and standard deviation: semi-open
mean(data$Job_satisfaction_semiopen1_5_sentimentr)
## [1] 3.221665
sd(data$Job_satisfaction_semiopen1_5_sentimentr)
## [1] 0.7565059
psych::alpha(data[,c('Job_satisfaction_semiopen1_sentimentr','Job_satisfaction_semiopen2_sentimentr', 'Job_satisfaction_semiopen3_sentimentr', 'Job_satisfaction_semiopen4_sentimentr', 'Job_satisfaction_semiopen5_sentimentr')])$total$raw_alpha
## [1] 0.7185338
```

### Wave 2

```
data_w2$Job_satisfaction_semiopen1_5_sentimentr <- fn(sentimentr::sentiment(text.var = as.character(data_w2$Job_satisfaction_semi1_5_text_LC))$sentiment)
data_w2$Job_satisfaction_semiopen1_sentimentr <- fn(sentimentr::sentiment(text.var = as.character(data_w2$Job_satisfaction_semi1_text))$sentiment)
data_w2$Job_satisfaction_semiopen2_sentimentr <- fn(sentimentr::sentiment(text.var = as.character(data_w2$Job_satisfaction_semi2_text))$sentiment)
data_w2$Job_satisfaction_semiopen3_sentimentr <- fn(sentimentr::sentiment(text.var = as.character(data_w2$Job_satisfaction_semi3_text))$sentiment)
data_w2$Job_satisfaction_semiopen4_sentimentr <- fn(sentimentr::sentiment(text.var = as.character(data_w2$Job_satisfaction_semi4_text))$sentiment)
data_w2$Job_satisfaction_semiopen5_sentimentr <- fn(sentimentr::sentiment(text.var = as.character(data_w2$Job_satisfaction_semi5_text))$sentiment)
data_w2$Job_satisfaction_open_sentimentr <- fn(sentimentr::sentiment(text.var = data_w2$Job_satisfaction_open_text_LC)$sentiment)

data_w2 <- mutate(data_w2, Job_satisfaction_semiopen4_sentimentr = ifelse(is.na(Semiopen_rating_4), NA, Job_satisfaction_semiopen4_sentimentr),Job_satisfaction_semiopen5_sentimentr = ifelse(is.na(Semiopen_rating_5), NA,Job_satisfaction_semiopen5_sentimentr))

#mean and standard deviation: open
mean(data_w2$Job_satisfaction_open_sentimentr)
## [1] 3.094828
sd(data_w2$Job_satisfaction_open_sentimentr)
## [1] 0.8338329

#mean and standard deviation: semi-open
mean(data_w2$Job_satisfaction_semiopen1_5_sentimentr)
## [1] 3.051724
sd(data_w2$Job_satisfaction_semiopen1_5_sentimentr)
## [1] 1.070106
psych::alpha(data_w2[,c('Job_satisfaction_semiopen1_sentimentr','Job_satisfaction_semiopen2_sentimentr', 'Job_satisfaction_semiopen3_sentimentr', 'Job_satisfaction_semiopen4_sentimentr', 'Job_satisfaction_semiopen5_sentimentr')])$total$raw_alpha
## [1] 0.6618776
```

## SentiStrength

### Wave 1

```
setwd(paste0(path, "/SentiStrength_results"))

SentiStrength_semiopen <- read.table("Semiopen_combined_study_1+results.txt", sep = "\t", header = TRUE)

Open_part1 <- read.table("Standalone_text_study1_checked1+results.txt", sep = "\t", header = TRUE)
Open_part2 <- read.table("Standalone_text_study1_checked2+results.txt", sep = "\t", header = TRUE)
SentiStrength_open <- bind_rows(Open_part1, Open_part2)

#data <- bind_cols(data, SentiStrength_open)
#data <- bind_cols(data, SentiStrength_semiopen)
SentiStrength_semiopen <- read.table("Semiopen_combined_study_1+results.txt", sep = "\t", header = TRUE)
SentiStrength_semiopen1 <- read.table("Semiopen1_study_1+results.txt", sep = "\t", header = TRUE)
SentiStrength_semiopen2 <- read.table("Semiopen2_study_1+results.txt", sep = "\t", header = TRUE)
SentiStrength_semiopen3 <- read.table("Semiopen3_study_1+results.txt", sep = "\t", header = TRUE)
SentiStrength_semiopen4 <- read.table("Semiopen4_study_1+results.txt", sep = "\t", header = TRUE)
SentiStrength_semiopen5 <- read.table("Semiopen5_study_1+results.txt", sep = "\t", header = TRUE)

data <- bind_cols(data, SentiStrength_open, SentiStrength_semiopen, SentiStrength_semiopen1, SentiStrength_semiopen2, SentiStrength_semiopen3, SentiStrength_semiopen4, SentiStrength_semiopen5) %>%
  rename(SentiStrength_open_neg = Negative, SentiStrength_open_pos = Positive, SentiStrength_semi_open_neg = Negative1, 
  SentiStrength_semi_open_pos = Positive1, SentiStrength_semi_open1_neg = Negative2, SentiStrength_semi_open1_pos = Positive2, SentiStrength_semi_open2_neg = Negative3, SentiStrength_semi_open2_pos = Positive3, SentiStrength_semi_open3_neg = Negative4,SentiStrength_semi_open3_pos = Positive4, SentiStrength_semi_open4_neg = Negative5, SentiStrength_semi_open4_pos = Positive5,  SentiStrength_semi_open5_neg = Negative6,SentiStrength_semi_open5_pos = Positive6)

data <- data %>% mutate(Job_satisfaction_open_sentistrength = fn(rowSums(cbind(SentiStrength_open_neg, SentiStrength_open_pos))),
 Job_satisfaction_semiopen1_5_sentistrength = fn(rowSums(cbind(SentiStrength_semi_open_neg,SentiStrength_semi_open_pos))),
 Job_satisfaction_semi_open1_sentistrength = fn(rowSums(cbind(SentiStrength_semi_open1_neg,SentiStrength_semi_open1_pos))),
 Job_satisfaction_semi_open2_sentistrength = fn(rowSums(cbind(SentiStrength_semi_open2_neg,SentiStrength_semi_open2_pos))),
 Job_satisfaction_semi_open3_sentistrength = fn(rowSums(cbind(SentiStrength_semi_open3_neg,SentiStrength_semi_open3_pos))),
 Job_satisfaction_semi_open4_sentistrength = fn(rowSums(cbind(SentiStrength_semi_open4_neg,SentiStrength_semi_open4_pos))),
 Job_satisfaction_semi_open5_sentistrength = fn(rowSums(cbind(SentiStrength_semi_open5_neg,SentiStrength_semi_open5_pos)))) %>%
  mutate(Job_satisfaction_semi_open4_sentistrength = ifelse(is.na(Resp_semi4), NA,
            Job_satisfaction_semi_open4_sentistrength),
         Job_satisfaction_semi_open5_sentistrength = ifelse(is.na(Resp_semi5), NA,
            Job_satisfaction_semi_open5_sentistrength))
#descriptives semi-open
mean(data$Job_satisfaction_semiopen1_5_sentistrength)
## [1] 3.410231
sd(data$Job_satisfaction_semiopen1_5_sentistrength)
## [1] 0.6849029
psych::alpha(data[,c('Job_satisfaction_semi_open1_sentistrength','Job_satisfaction_semi_open2_sentistrength', 'Job_satisfaction_semi_open3_sentistrength', 'Job_satisfaction_semi_open4_sentistrength', 'Job_satisfaction_semi_open5_sentistrength')])$total$raw_alpha
## [1] 0.6689317

# descrptives open
mean(data$Job_satisfaction_open_sentistrength)
## [1] 3.091274
sd(data$Job_satisfaction_open_sentistrength)
## [1] 0.7244262
```

### Wave 2

```
setwd(paste0(path, "/SentiStrength_results"))

SentiStrength_semiopen <- read.table("Semiopen_combined_study_1_w2+results.txt", sep = "\t", header = TRUE)
SentiStrength_semiopen1 <- read.table("Semiopen1_study_1_w2+results.txt", sep = "\t", header = TRUE)
SentiStrength_semiopen2 <- read.table("Semiopen2_study_1_w2+results.txt", sep = "\t", header = TRUE)
SentiStrength_semiopen3 <- read.table("Semiopen3_study_1_w2+results.txt", sep = "\t", header = TRUE)
SentiStrength_semiopen4 <- read.table("Semiopen4_study_1_w2+results.txt", sep = "\t", header = TRUE)
SentiStrength_semiopen5 <- read.table("Semiopen5_study_1_w2+results.txt", sep = "\t", header = TRUE)

SentiStrength_open <- read.table("Standalone_text_study1_checked1_w2+results.txt", sep = "\t", header = TRUE)

data_w2 <- bind_cols(data_w2, SentiStrength_open, SentiStrength_semiopen, SentiStrength_semiopen1, 
     SentiStrength_semiopen2, SentiStrength_semiopen3, 
     SentiStrength_semiopen4, SentiStrength_semiopen5) %>%
  rename(SentiStrength_open_neg = Negative, 
  SentiStrength_open_pos = Positive, 
  SentiStrength_semi_open_neg = Negative1, 
  SentiStrength_semi_open_pos = Positive1,
  SentiStrength_semi_open1_neg = Negative2,
  SentiStrength_semi_open1_pos = Positive2,
  SentiStrength_semi_open2_neg = Negative3,
  SentiStrength_semi_open2_pos = Positive3,
  SentiStrength_semi_open3_neg = Negative4,
  SentiStrength_semi_open3_pos = Positive4,
  SentiStrength_semi_open4_neg = Negative5,
  SentiStrength_semi_open4_pos = Positive5,
  SentiStrength_semi_open5_neg = Negative6,
  SentiStrength_semi_open5_pos = Positive6) %>%
  mutate(Job_satisfaction_open_sentistrength = fn(rowSums(cbind(SentiStrength_open_neg,SentiStrength_open_pos))),
 Job_satisfaction_semiopen1_5_sentistrength = fn(rowSums(cbind(SentiStrength_semi_open_neg,SentiStrength_semi_open_pos))),
 Job_satisfaction_semi_open1_sentistrength = fn(rowSums(cbind(SentiStrength_semi_open1_neg,SentiStrength_semi_open1_pos))),
 Job_satisfaction_semi_open2_sentistrength = fn(rowSums(cbind(SentiStrength_semi_open2_neg,SentiStrength_semi_open1_pos))),
 Job_satisfaction_semi_open3_sentistrength = fn(rowSums(cbind(SentiStrength_semi_open3_neg,SentiStrength_semi_open1_pos))),
 Job_satisfaction_semi_open4_sentistrength = fn(rowSums(cbind(SentiStrength_semi_open4_neg,SentiStrength_semi_open1_pos))),
 Job_satisfaction_semi_open5_sentistrength = fn(rowSums(cbind(SentiStrength_semi_open5_neg,SentiStrength_semi_open1_pos)))) %>%
  mutate(Job_satisfaction_semi_open4_sentistrength = ifelse(is.na(Semiopen_rating_4), NA,
            Job_satisfaction_semi_open4_sentistrength),
         Job_satisfaction_semi_open5_sentistrength = ifelse(is.na(Semiopen_rating_5), NA,
            Job_satisfaction_semi_open5_sentistrength))
         

#descriptives semi-open
mean(data_w2$Job_satisfaction_semiopen1_5_sentistrength)
## [1] 3.258621
sd(data_w2$Job_satisfaction_semiopen1_5_sentistrength)
## [1] 0.7815335
psych::alpha(data_w2[,c('Job_satisfaction_semi_open1_sentistrength','Job_satisfaction_semi_open2_sentistrength', 'Job_satisfaction_semi_open3_sentistrength', 'Job_satisfaction_semi_open4_sentistrength', 'Job_satisfaction_semi_open5_sentistrength')])$total$raw_alpha
## [1] 0.9590177

# descrptives open
mean(data_w2$Job_satisfaction_open_sentistrength)
## [1] 3.163793
sd(data_w2$Job_satisfaction_open_sentistrength)
## [1] 0.9226047
```

## Summary of ratings

```
data_selec <- data %>% select(Job_satisfaction_semiopen1_5_LIWC, Job_satisfaction_open_LIWC,
#sentiStrength
Job_satisfaction_semiopen1_5_sentistrength, Job_satisfaction_open_sentistrength,
#sentimentr
Job_satisfaction_semiopen1_5_sentimentr, Job_satisfaction_open_sentimentr,   
#independent raters 
Job_satisfaction_semiopen1_5_raters, Job_satisfaction_open_raters, 
#closed question
Job_satisfaction_closed)
dataw2_selec <- data_w2 %>% select(Job_satisfaction_semiopen1_5_LIWC_w2, Job_satisfaction_open_LIWC_w2,
#sentiStrength
Job_satisfaction_semiopen1_5_sentistrength, Job_satisfaction_open_sentistrength,
#sentimentr
Job_satisfaction_semiopen1_5_sentimentr, Job_satisfaction_open_sentimentr,   
#independent raters 
Job_satisfaction_semiopen1_5_raters, Job_satisfaction_open_raters, 
#closed question
Job_satisfaction_closed) %>%
  rename(Job_satisfaction_open_LIWC = Job_satisfaction_open_LIWC_w2, 
         Job_satisfaction_semiopen1_5_LIWC = Job_satisfaction_semiopen1_5_LIWC_w2 )
  

data_combined <- rbind(dataw2_selec, data_selec)
```

```
df_semi <- data_combined %>%
  select(Job_satisfaction_semiopen1_5_raters, 
         Job_satisfaction_semiopen1_5_LIWC, Job_satisfaction_semiopen1_5_sentimentr, 
         Job_satisfaction_semiopen1_5_sentistrength) %>%
  rename(`Independent raters` = Job_satisfaction_semiopen1_5_raters,
         `LIWC 2015` = Job_satisfaction_semiopen1_5_LIWC,
         `SentimentR` = Job_satisfaction_semiopen1_5_sentimentr,
         `SentiStrength` = Job_satisfaction_semiopen1_5_sentistrength) %>%
  lapply(function(x) fn(x))

df_semi <- as.data.frame(df_semi)
tiff("semi_open.tiff", units="in", width=5, height=5, res=400)

df_semi %>%
  gather() %>% 
  ggplot(aes(value)) +
    facet_wrap(~ key, scales = "free") +
    geom_histogram() +
  scale_x_continuous(name = 'Score') +
  scale_y_continuous(name = 'Frequency') + theme_bw() + theme(strip.text.x = element_text(size = 12), axis.text.x = element_text(size = 12), axis.text.y = element_text(size = 12))
```

```
## `stat_bin()` using `bins = 30`. Pick better value with `binwidth`.
```

```
dev.off()
```

```
## png 
##   2
```

```
df_open <- data_combined %>%
  select(Job_satisfaction_open_raters, Job_satisfaction_open_LIWC, Job_satisfaction_open_sentimentr, Job_satisfaction_open_sentistrength) %>%
  rename(`Independent raters` = Job_satisfaction_open_raters,
         `LIWC 2015` = Job_satisfaction_open_LIWC,
         `SentimentR` = Job_satisfaction_open_sentimentr,
         `SentiStrength` = Job_satisfaction_open_sentistrength)  %>%
  lapply(function(x) fn(x))
df_open <- as.data.frame(df_open)

tiff("open.tiff", units="in", width=5, height=5, res=400)

df_open %>%
  gather() %>% 
  ggplot(aes(value)) +
    facet_wrap(~ key, scales = "free") +
    geom_histogram() +
  scale_x_continuous(name = 'Score') +
  scale_y_continuous(name = 'Frequency') + theme_bw() + theme(strip.text.x = element_text(size = 12), axis.text.x = element_text(size = 12), axis.text.y = element_text(size = 12))
```

```
## `stat_bin()` using `bins = 30`. Pick better value with `binwidth`.
```

```
dev.off()
```

```
## png 
##   2
```

# Descriptive statistics

## Wave 1

### Descriptive statistics survey variables

```
Education <- data.frame(ftable(data$Education))
Education$pct <- round(x = (Education$Freq/sum(Education$Freq))*100, digits = 2)
Education
##                                                          Var1 Freq   pct
## 1                                        {"ImportId":"QID12"}    0  0.00
## 2                                               2 year degree   79  7.92
## 3                                               4 year degree  278 27.88
## 4                                                   Doctorate   22  2.21
## 5                                        High school graduate  139 13.94
## 6                                       Less than high school    8  0.80
## 7                                              Masters degree  126 12.64
## 8                                         Professional degree   87  8.73
## 9                                                Some college  258 25.88
## 10 What is the highest level of education you have completed?    0  0.00

Gender <- data.frame(ftable(data$Gender))
Gender$pct <- round(x = (Gender$Freq/sum(Gender$Freq))*100, digits = 2)
Gender
##                   Var1 Freq   pct
## 1  {"ImportId":"QID8"}    0  0.00
## 2               Female  744 74.62
## 3                 Male  253 25.38
## 4 What is your gender?    0  0.00

Age <- data.frame(statistic = c('Mean', 'Stdv'),
  score = c(round(x = mean(as.numeric.factor(data$Age), na.rm = TRUE), digits = 2),round(x = sd(as.numeric.factor(data$Age), na.rm = TRUE), digits = 2)))
Age
##   statistic score
## 1      Mean 35.59
## 2      Stdv  9.80

Marital_status <- data.frame(ftable(data$Marital_status))
Marital_status$pct <- round(x = (Marital_status$Freq/sum(Marital_status$Freq))*100, digits = 2)
Marital_status
##                           Var1 Freq   pct
## 1          {"ImportId":"QID9"}    0  0.00
## 2                     Divorced   45  4.51
## 3            In a relationship  317 31.80
## 4                      Married  446 44.73
## 5                       Single  185 18.56
## 6 What is your marital status?    0  0.00
## 7                      Widowed    4  0.40

round(mean(as.numeric(as.character(data$Satisfaction_work_content)), na.rm = TRUE), 2)
## [1] 6.56
round(sd(as.numeric(as.character(data$Satisfaction_work_content)), na.rm = TRUE), 2)
## [1] 2.26

round(mean(as.numeric(as.character(data$Satisfaction_work_environ)), na.rm = TRUE), 2)
## [1] 6.61
round(sd(as.numeric(as.character(data$Satisfaction_work_environ)), na.rm = TRUE), 2)
## [1] 2.25

round(mean(as.numeric(as.character(data$Satisfaction_team)), na.rm = TRUE), 2)
## [1] 7.45
round(sd(as.numeric(as.character(data$Satisfaction_team)), na.rm = TRUE), 2)
## [1] 2.08

round(mean(as.numeric(as.character(data$Satisfaction_supervisor)), na.rm = TRUE), 2)
## [1] 6.77
round(sd(as.numeric(as.character(data$Satisfaction_supervisor)), na.rm = TRUE), 2)
## [1] 2.79

round(mean(as.numeric(as.character(data$Satisfaction_work_private)), na.rm = TRUE), 2)
## [1] 6.45
round(sd(as.numeric(as.character(data$Satisfaction_work_private)), na.rm = TRUE), 2)
## [1] 2.49

round(mean(as.numeric(as.character(data$Satisfaction_company)), na.rm = TRUE), 2)
## [1] 6.47
round(sd(as.numeric(as.character(data$Satisfaction_company)), na.rm = TRUE), 2)
## [1] 2.52

round(mean(as.numeric(as.character(data$Satisfaction_pay)), na.rm = TRUE), 2)
## [1] 5.44
round(sd(as.numeric(as.character(data$Satisfaction_pay)), na.rm = TRUE), 2)
## [1] 2.47

round(mean(as.numeric(as.character(data$Job_satisfaction_closed)), na.rm = TRUE), 2)
## [1] 6.42
round(sd(as.numeric(as.character(data$Job_satisfaction_closed)), na.rm = TRUE), 2)
## [1] 2.37
```

### Creating frequency tables

Based on the general job satisfaction question, we created three groups: dissatisfied, neutral and satisfied. We subsetted data based on this classification.

```
data$Job_satisfaction_closed <- as.numeric(as.character(data$Job_satisfaction_closed))
negative <- filter(data, Job_satisfaction_closed <= 4) %>% select(Job_satisfaction_semi1_5_text_LC, Job_satisfaction_open_text_LC)
neutral <- filter(data, Job_satisfaction_closed  >= 5 & Job_satisfaction_closed <= 6) %>% select(Job_satisfaction_semi1_5_text_LC, Job_satisfaction_open_text_LC)
positive <- filter(data, Job_satisfaction_closed >= 7) %>% select(Job_satisfaction_semi1_5_text_LC, Job_satisfaction_open_text_LC)
```

```
#semi-open
words <- Corpus(VectorSource(negative$Job_satisfaction_semi1_5_text_LC))
text_doc <- as.matrix(TermDocumentMatrix(words))
v <- sort(rowSums(text_doc), decreasing =  TRUE)
b <- data.frame(word = names(v), freq =  v)
head(b, 10)
##                    word freq
## boring           boring   56
## stressful     stressful   41
## repetitive   repetitive   29
## tiring           tiring   25
## busy               busy   19
## frustrating frustrating   19
## challenging challenging   16
## hard               hard   16
## rewarding     rewarding   13
## dull               dull   13

#open
words <- Corpus(VectorSource(negative$Job_satisfaction_open_text_LC))
words <- tm_map(words, removeWords, stopwords("english"))
text_doc <- as.matrix(TermDocumentMatrix(words))
v <- sort(rowSums(text_doc), decreasing =  TRUE)
b <- data.frame(word = names(v), freq =  v)
head(b, 10)
##            word freq
## job         job  244
## work       work  207
## feel       feel  111
## like       like  109
## enjoy     enjoy   53
## get         get   53
## time       time   51
## people   people   50
## much       much   38
## however however   37
```

```
#Semi-open
words <- Corpus(VectorSource(neutral$Job_satisfaction_semi1_5_text_LC))
text_doc <- as.matrix(TermDocumentMatrix(words))
v <- sort(rowSums(text_doc), decreasing =  TRUE)
b <- data.frame(word = names(v), freq =  v)
head(b, 10)
##                    word freq
## rewarding     rewarding   39
## challenging challenging   35
## stressful     stressful   31
## busy               busy   30
## interesting interesting   27
## boring           boring   26
## hard               hard   25
## tiring           tiring   25
## repetitive   repetitive   23
## easy               easy   18

#open
words <- Corpus(VectorSource(neutral$Job_satisfaction_open_text_LC))
words <- tm_map(words, removeWords, stopwords("english"))
text_doc <- as.matrix(TermDocumentMatrix(words))
v <- sort(rowSums(text_doc), decreasing =  TRUE)
b <- data.frame(word = names(v), freq =  v)
head(b, 10)
##          word freq
## job       job  282
## work     work  235
## feel     feel  133
## like     like  105
## enjoy   enjoy   83
## get       get   67
## happy   happy   65
## people people   64
## can       can   58
## time     time   49
```

```
#Semi-open
words <- Corpus(VectorSource(positive$Job_satisfaction_semi1_5_text_LC))
text_doc <- as.matrix(TermDocumentMatrix(words))
v <- sort(rowSums(text_doc), decreasing =  TRUE)
b <- data.frame(word = names(v), freq =  v)
head(b, 10)
##                    word freq
## rewarding     rewarding  131
## challenging challenging  103
## interesting interesting   97
## busy               busy   79
## fun                 fun   73
## important     important   50
## stressful     stressful   44
## happy             happy   42
## enjoyable     enjoyable   41
## exciting       exciting   40

#open
words <- Corpus(VectorSource(positive$Job_satisfaction_open_text_LC))
words <- tm_map(words, removeWords, stopwords("english"))
text_doc <- as.matrix(TermDocumentMatrix(words))
v <- sort(rowSums(text_doc), decreasing =  TRUE)
b <- data.frame(word = names(v), freq =  v)
head(b, 10)
##            word freq
## job         job  650
## work       work  546
## feel       feel  326
## happy     happy  248
## like       like  245
## enjoy     enjoy  224
## working working  141
## can         can  131
## people   people  118
## get         get  106
```

## Wave 2

### Descriptive statistics survey variables

```
ids_w2 <- data_w2$ProlificID

data_w2_1 <- subset(data, ProlificID %in% ids_w2)

Education <- data.frame(ftable(data_w2_1$Education))
Education$pct <- round(x = (Education$Freq/sum(Education$Freq))*100, digits = 2)
Education
##                                                          Var1 Freq   pct
## 1                                        {"ImportId":"QID12"}    0  0.00
## 2                                               2 year degree   13 11.21
## 3                                               4 year degree   40 34.48
## 4                                                   Doctorate    2  1.72
## 5                                        High school graduate   13 11.21
## 6                                       Less than high school    1  0.86
## 7                                              Masters degree   20 17.24
## 8                                         Professional degree    4  3.45
## 9                                                Some college   23 19.83
## 10 What is the highest level of education you have completed?    0  0.00

Gender <- data.frame(ftable(data_w2_1$Gender))
Gender$pct <- round(x = (Gender$Freq/sum(Gender$Freq))*100, digits = 2)
Gender
##                   Var1 Freq   pct
## 1  {"ImportId":"QID8"}    0  0.00
## 2               Female   68 58.62
## 3                 Male   48 41.38
## 4 What is your gender?    0  0.00

Age <- data.frame(statistic = c('Mean', 'Stdv'),
  score = c(round(x = mean(as.numeric.factor(data_w2_1$Age), na.rm = TRUE), digits = 2),round(x = sd(as.numeric.factor(data_w2_1$Age), na.rm = TRUE), digits = 2)))
Age
##   statistic score
## 1      Mean 39.71
## 2      Stdv 10.51

Marital_status <- data.frame(ftable(data_w2_1$Marital_status))
Marital_status$pct <- round(x = (Marital_status$Freq/sum(Marital_status$Freq))*100, digits = 2)
Marital_status
##                           Var1 Freq   pct
## 1          {"ImportId":"QID9"}    0  0.00
## 2                     Divorced    9  7.76
## 3            In a relationship   29 25.00
## 4                      Married   55 47.41
## 5                       Single   23 19.83
## 6 What is your marital status?    0  0.00
## 7                      Widowed    0  0.00

round(mean(as.numeric(as.character(data_w2$Job_satisfaction_closed)), na.rm = TRUE), 2)
## [1] 5.99
round(sd(as.numeric(as.character(data_w2$Job_satisfaction_closed)), na.rm = TRUE), 2)
## [1] 2.91
```

# Hypothesis testing

## Specific factor error

```
data[,c( 'Satisfaction_work_private', 'Satisfaction_work_content', 'Satisfaction_supervisor', 'Satisfaction_company', 'Satisfaction_team', 'Satisfaction_work_environ', 'Satisfaction_pay')] <- as.numeric(as.character(unlist(data[,c( 'Satisfaction_work_private', 'Satisfaction_work_content', 'Satisfaction_supervisor', 'Satisfaction_company', 'Satisfaction_team', 'Satisfaction_work_environ', 'Satisfaction_pay')])))


data_combined$Job_satisfaction_closed <- as.numeric(as.character(data_combined$Job_satisfaction_closed))

specific_factor_semi_open <- round(cor(x = as.matrix(data_combined[, c('Job_satisfaction_semiopen1_5_raters',
 'Job_satisfaction_semiopen1_5_LIWC', 
 'Job_satisfaction_semiopen1_5_sentimentr',
 'Job_satisfaction_semiopen1_5_sentistrength',
 'Job_satisfaction_closed')])), 3)
specific_factor_semi_open
##                                            Job_satisfaction_semiopen1_5_raters
## Job_satisfaction_semiopen1_5_raters                                      1.000
## Job_satisfaction_semiopen1_5_LIWC                                        0.775
## Job_satisfaction_semiopen1_5_sentimentr                                  0.772
## Job_satisfaction_semiopen1_5_sentistrength                               0.696
## Job_satisfaction_closed                                                  0.628
##                                            Job_satisfaction_semiopen1_5_LIWC
## Job_satisfaction_semiopen1_5_raters                                    0.775
## Job_satisfaction_semiopen1_5_LIWC                                      1.000
## Job_satisfaction_semiopen1_5_sentimentr                                0.708
## Job_satisfaction_semiopen1_5_sentistrength                             0.704
## Job_satisfaction_closed                                                0.576
##                                            Job_satisfaction_semiopen1_5_sentimentr
## Job_satisfaction_semiopen1_5_raters                                          0.772
## Job_satisfaction_semiopen1_5_LIWC                                            0.708
## Job_satisfaction_semiopen1_5_sentimentr                                      1.000
## Job_satisfaction_semiopen1_5_sentistrength                                   0.665
## Job_satisfaction_closed                                                      0.593
##                                            Job_satisfaction_semiopen1_5_sentistrength
## Job_satisfaction_semiopen1_5_raters                                             0.696
## Job_satisfaction_semiopen1_5_LIWC                                               0.704
## Job_satisfaction_semiopen1_5_sentimentr                                         0.665
## Job_satisfaction_semiopen1_5_sentistrength                                      1.000
## Job_satisfaction_closed                                                         0.547
##                                            Job_satisfaction_closed
## Job_satisfaction_semiopen1_5_raters                          0.628
## Job_satisfaction_semiopen1_5_LIWC                            0.576
## Job_satisfaction_semiopen1_5_sentimentr                      0.593
## Job_satisfaction_semiopen1_5_sentistrength                   0.547
## Job_satisfaction_closed                                      1.000

specific_factor_open <- round(cor(x = as.matrix(data_combined[, c(
 'Job_satisfaction_open_raters',
 'Job_satisfaction_open_LIWC', 
 'Job_satisfaction_open_sentimentr',
 'Job_satisfaction_open_sentistrength', 
 'Job_satisfaction_closed')])), 3)
specific_factor_open
##                                     Job_satisfaction_open_raters
## Job_satisfaction_open_raters                               1.000
## Job_satisfaction_open_LIWC                                 0.508
## Job_satisfaction_open_sentimentr                           0.532
## Job_satisfaction_open_sentistrength                        0.587
## Job_satisfaction_closed                                    0.726
##                                     Job_satisfaction_open_LIWC
## Job_satisfaction_open_raters                             0.508
## Job_satisfaction_open_LIWC                               1.000
## Job_satisfaction_open_sentimentr                         0.512
## Job_satisfaction_open_sentistrength                      0.510
## Job_satisfaction_closed                                  0.393
##                                     Job_satisfaction_open_sentimentr
## Job_satisfaction_open_raters                                   0.532
## Job_satisfaction_open_LIWC                                     0.512
## Job_satisfaction_open_sentimentr                               1.000
## Job_satisfaction_open_sentistrength                            0.487
## Job_satisfaction_closed                                        0.407
##                                     Job_satisfaction_open_sentistrength
## Job_satisfaction_open_raters                                      0.587
## Job_satisfaction_open_LIWC                                        0.510
## Job_satisfaction_open_sentimentr                                  0.487
## Job_satisfaction_open_sentistrength                               1.000
## Job_satisfaction_closed                                           0.464
##                                     Job_satisfaction_closed
## Job_satisfaction_open_raters                          0.726
## Job_satisfaction_open_LIWC                            0.393
## Job_satisfaction_open_sentimentr                      0.407
## Job_satisfaction_open_sentistrength                   0.464
## Job_satisfaction_closed                               1.000
#Hypothesis 1a

fisherz2r(mean(fisherz(.508),fisherz(.532),fisherz(.587)))
## [1] 0.508
fisherz2r(mean(fisherz(.774),fisherz(.772),fisherz(.695)))
## [1] 0.774

t.test(x = c(fisherz(.508),fisherz(.532),fisherz(.587)), y = c(fisherz(.774),fisherz(.772),fisherz(.695)))
## 
##  Welch Two Sample t-test
## 
## data:  c(fisherz(0.508), fisherz(0.532), fisherz(0.587)) and c(fisherz(0.774), fisherz(0.772), fisherz(0.695))
## t = -5.4957, df = 3.2472, p-value = 0.0096
## alternative hypothesis: true difference in means is not equal to 0
## 95 percent confidence interval:
##  -0.5634087 -0.1612670
## sample estimates:
## mean of x mean of y 
## 0.6086792 0.9710170

#hypothesis 1b
##independent - SentiStrength vs LIWC
paired.r(.587,.508, .510, 1116)
## Call: paired.r(xy = 0.587, xz = 0.508, yz = 0.51, n = 1116)
## [1] "test of difference between two correlated  correlations"
## t = 3.38  With probability =  0

##independent - SentiStrength vs Sentimentr
paired.r(.587,.532, .487, 1116)
## Call: paired.r(xy = 0.587, xz = 0.532, yz = 0.487, n = 1116)
## [1] "test of difference between two correlated  correlations"
## t = 2.33  With probability =  0.02

##independent - LIWC vs Sentimentr
paired.r(.532, .508, .512, 1116)
## Call: paired.r(xy = 0.532, xz = 0.508, yz = 0.512, n = 1116)
## [1] "test of difference between two correlated  correlations"
## t = 1  With probability =  0.32

#hypothesis 1c
##independent - LIWC vs SentiStrength
paired.r(.774,.695, .704, 1116)
## Call: paired.r(xy = 0.774, xz = 0.695, yz = 0.704, n = 1116)
## [1] "test of difference between two correlated  correlations"
## t = 5.63  With probability =  0

##independent - SentimentR vs SentiStrength
paired.r(.772,.695, .665, 1116)
## Call: paired.r(xy = 0.772, xz = 0.695, yz = 0.665, n = 1116)
## [1] "test of difference between two correlated  correlations"
## t = 5.21  With probability =  0

##independent - LIWC vs Sentimentr
paired.r(.772, .774, .708, 1116)
## Call: paired.r(xy = 0.772, xz = 0.774, yz = 0.708, n = 1116)
## [1] "test of difference between two correlated  correlations"
## t = -0.16  With probability =  0.88
```

## Algorithm error

```
#semi
kripp.alpha(t(data.frame(x = data_combined$Job_satisfaction_semiopen1_5_LIWC, y = data_combined$Job_satisfaction_semiopen1_5_sentimentr, z = data_combined$Job_satisfaction_semiopen1_5_sentistrength)), method = 'interval')
##  Krippendorff's alpha
## 
##  Subjects = 1113 
##    Raters = 3 
##     alpha = 0.506

kripp.alpha(t(data.frame(x = data_combined$Job_satisfaction_semiopen1_5_LIWC, y = data_combined$Job_satisfaction_semiopen1_5_sentimentr)), method = 'interval')
##  Krippendorff's alpha
## 
##  Subjects = 1113 
##    Raters = 2 
##     alpha = 0.495

kripp.alpha(t(data.frame(x = data_combined$Job_satisfaction_semiopen1_5_LIWC, y = data_combined$Job_satisfaction_semiopen1_5_sentistrength)), method = 'interval')
##  Krippendorff's alpha
## 
##  Subjects = 1113 
##    Raters = 2 
##     alpha = 0.471

kripp.alpha(t(data.frame( y = data_combined$Job_satisfaction_semiopen1_5_sentimentr, z = data_combined$Job_satisfaction_semiopen1_5_sentistrength)), method = 'interval')
##  Krippendorff's alpha
## 
##  Subjects = 1113 
##    Raters = 2 
##     alpha = 0.633

#open
kripp.alpha(t(data.frame(x = data_combined$Job_satisfaction_open_LIWC, y = data_combined$Job_satisfaction_open_sentimentr, z = data_combined$Job_satisfaction_open_sentistrength)), method = 'interval')
##  Krippendorff's alpha
## 
##  Subjects = 1113 
##    Raters = 3 
##     alpha = 0.187

kripp.alpha(t(data.frame(y = data_combined$Job_satisfaction_open_sentimentr, z = data_combined$Job_satisfaction_open_sentistrength)), method = 'interval')
##  Krippendorff's alpha
## 
##  Subjects = 1113 
##    Raters = 2 
##     alpha = 0.434

kripp.alpha(t(data.frame(x = data_combined$Job_satisfaction_open_LIWC,z = data_combined$Job_satisfaction_open_sentistrength)), method = 'interval')
##  Krippendorff's alpha
## 
##  Subjects = 1113 
##    Raters = 2 
##     alpha = 0.159

kripp.alpha(t(data.frame(x = data_combined$Job_satisfaction_open_LIWC, y = data_combined$Job_satisfaction_open_sentimentr)), method = 'interval')
##  Krippendorff's alpha
## 
##  Subjects = 1113 
##    Raters = 2 
##     alpha = 0.0102
```

## Transient error

```
ids_repeat <- data_w2$ProlificID

data_t_retest <- data %>% filter(ProlificID %in% ids_repeat) %>% select(c(Job_satisfaction_semiopen1_5_raters, 
         Job_satisfaction_semiopen1_5_LIWC, Job_satisfaction_semiopen1_5_sentimentr, 
         Job_satisfaction_semiopen1_5_sentistrength, Job_satisfaction_closed,Job_satisfaction_semiopen1_5_LIWC,
         Job_satisfaction_open_raters, Job_satisfaction_open_LIWC, Job_satisfaction_open_sentimentr, Job_satisfaction_open_sentistrength,
         ProlificID))

data_t_retest <- left_join(data_t_retest,data_w2, by = "ProlificID")

cor(data_t_retest$Job_satisfaction_semiopen1_5_sentimentr.x, data_t_retest$Job_satisfaction_semiopen1_5_sentimentr.y)
## [1] 0.2444701
cor(data_t_retest$Job_satisfaction_semiopen1_5_sentistrength.x, data_t_retest$Job_satisfaction_semiopen1_5_sentistrength.y)
## [1] 0.2496772
cor(data_t_retest$Job_satisfaction_semiopen1_5_LIWC, data_t_retest$Job_satisfaction_semiopen1_5_LIWC_w2)
## [1] 0.3111295
cor(data_t_retest$Job_satisfaction_semiopen1_5_raters.x, data_t_retest$Job_satisfaction_semiopen1_5_raters.y)
## [1] 0.3123909
cor(data_t_retest$Job_satisfaction_closed.x, as.numeric.factor(data_t_retest$Job_satisfaction_closed.y))
## [1] 0.5021596

cor(data_t_retest$Job_satisfaction_open_sentimentr.x, data_t_retest$Job_satisfaction_open_sentimentr.y)
## [1] 0.3288632
cor(data_t_retest$Job_satisfaction_open_sentistrength.x, data_t_retest$Job_satisfaction_open_sentistrength.y)
## [1] 0.189512
cor(data_t_retest$Job_satisfaction_open_LIWC, data_t_retest$Job_satisfaction_open_LIWC_w2)
## [1] 0.2487816
cor(data_t_retest$Job_satisfaction_open_raters.x, data_t_retest$Job_satisfaction_open_raters.y)
## [1] 0.5427461
cor(data_t_retest$Job_satisfaction_closed.x, as.numeric.factor(data_t_retest$Job_satisfaction_closed.y))
## [1] 0.5021596
```

## Correlations for validity

We correlated the text-based measures with the closed questions measuring job satisfaction.

```
cur <- round(cor(y = as.matrix(data[,c('Job_satisfaction_closed','Satisfaction_work_environ', 'Satisfaction_work_content', 'Satisfaction_team', "Satisfaction_supervisor", "Satisfaction_work_private", "Satisfaction_company", "Satisfaction_pay")]), 
    x = as.matrix(data[, c(
 'Job_satisfaction_open_raters','Job_satisfaction_semiopen1_5_raters',
 'Job_satisfaction_open_LIWC','Job_satisfaction_semiopen1_5_LIWC', 
 'Job_satisfaction_open_sentimentr','Job_satisfaction_semiopen1_5_sentimentr',
 'Job_satisfaction_open_sentistrength','Job_satisfaction_semiopen1_5_sentistrength')])), 3)
cur
##                                            Job_satisfaction_closed
## Job_satisfaction_open_raters                                 0.703
## Job_satisfaction_semiopen1_5_raters                          0.618
## Job_satisfaction_open_LIWC                                   0.373
## Job_satisfaction_semiopen1_5_LIWC                            0.564
## Job_satisfaction_open_sentimentr                             0.382
## Job_satisfaction_semiopen1_5_sentimentr                      0.579
## Job_satisfaction_open_sentistrength                          0.457
## Job_satisfaction_semiopen1_5_sentistrength                   0.541
##                                            Satisfaction_work_environ
## Job_satisfaction_open_raters                                   0.503
## Job_satisfaction_semiopen1_5_raters                            0.458
## Job_satisfaction_open_LIWC                                     0.303
## Job_satisfaction_semiopen1_5_LIWC                              0.412
## Job_satisfaction_open_sentimentr                               0.301
## Job_satisfaction_semiopen1_5_sentimentr                        0.444
## Job_satisfaction_open_sentistrength                            0.374
## Job_satisfaction_semiopen1_5_sentistrength                     0.402
##                                            Satisfaction_work_content
## Job_satisfaction_open_raters                                   0.598
## Job_satisfaction_semiopen1_5_raters                            0.555
## Job_satisfaction_open_LIWC                                     0.333
## Job_satisfaction_semiopen1_5_LIWC                              0.497
## Job_satisfaction_open_sentimentr                               0.345
## Job_satisfaction_semiopen1_5_sentimentr                        0.511
## Job_satisfaction_open_sentistrength                            0.396
## Job_satisfaction_semiopen1_5_sentistrength                     0.480
##                                            Satisfaction_team
## Job_satisfaction_open_raters                           0.445
## Job_satisfaction_semiopen1_5_raters                    0.361
## Job_satisfaction_open_LIWC                             0.230
## Job_satisfaction_semiopen1_5_LIWC                      0.329
## Job_satisfaction_open_sentimentr                       0.256
## Job_satisfaction_semiopen1_5_sentimentr                0.367
## Job_satisfaction_open_sentistrength                    0.288
## Job_satisfaction_semiopen1_5_sentistrength             0.348
##                                            Satisfaction_supervisor
## Job_satisfaction_open_raters                                 0.512
## Job_satisfaction_semiopen1_5_raters                          0.438
## Job_satisfaction_open_LIWC                                   0.244
## Job_satisfaction_semiopen1_5_LIWC                            0.376
## Job_satisfaction_open_sentimentr                             0.305
## Job_satisfaction_semiopen1_5_sentimentr                      0.423
## Job_satisfaction_open_sentistrength                          0.300
## Job_satisfaction_semiopen1_5_sentistrength                   0.368
##                                            Satisfaction_work_private
## Job_satisfaction_open_raters                                   0.412
## Job_satisfaction_semiopen1_5_raters                            0.370
## Job_satisfaction_open_LIWC                                     0.233
## Job_satisfaction_semiopen1_5_LIWC                              0.309
## Job_satisfaction_open_sentimentr                               0.245
## Job_satisfaction_semiopen1_5_sentimentr                        0.328
## Job_satisfaction_open_sentistrength                            0.286
## Job_satisfaction_semiopen1_5_sentistrength                     0.288
##                                            Satisfaction_company
## Job_satisfaction_open_raters                              0.582
## Job_satisfaction_semiopen1_5_raters                       0.537
## Job_satisfaction_open_LIWC                                0.337
## Job_satisfaction_semiopen1_5_LIWC                         0.474
## Job_satisfaction_open_sentimentr                          0.342
## Job_satisfaction_semiopen1_5_sentimentr                   0.504
## Job_satisfaction_open_sentistrength                       0.368
## Job_satisfaction_semiopen1_5_sentistrength                0.475
##                                            Satisfaction_pay
## Job_satisfaction_open_raters                          0.355
## Job_satisfaction_semiopen1_5_raters                   0.336
## Job_satisfaction_open_LIWC                            0.200
## Job_satisfaction_semiopen1_5_LIWC                     0.301
## Job_satisfaction_open_sentimentr                      0.229
## Job_satisfaction_semiopen1_5_sentimentr               0.277
## Job_satisfaction_open_sentistrength                   0.199
## Job_satisfaction_semiopen1_5_sentistrength            0.282
```

```
#sentistrenght vs LIWC convergence - human coding
paired.r(.775,.696, .704, 1116)
```

```
## Call: paired.r(xy = 0.775, xz = 0.696, yz = 0.704, n = 1116)
## [1] "test of difference between two correlated  correlations"
## t = 5.64  With probability =  0
```
