# Supplementary figures and images for: The promise of open survey questions—The validation of text-based job satisfaction measures
Source: PLoS One. 2019 Dec 26;14(12):e0226408. doi: 10.1371/journal.pone.0226408 (PMC6932814; doi:10.1371/journal.pone.0226408)

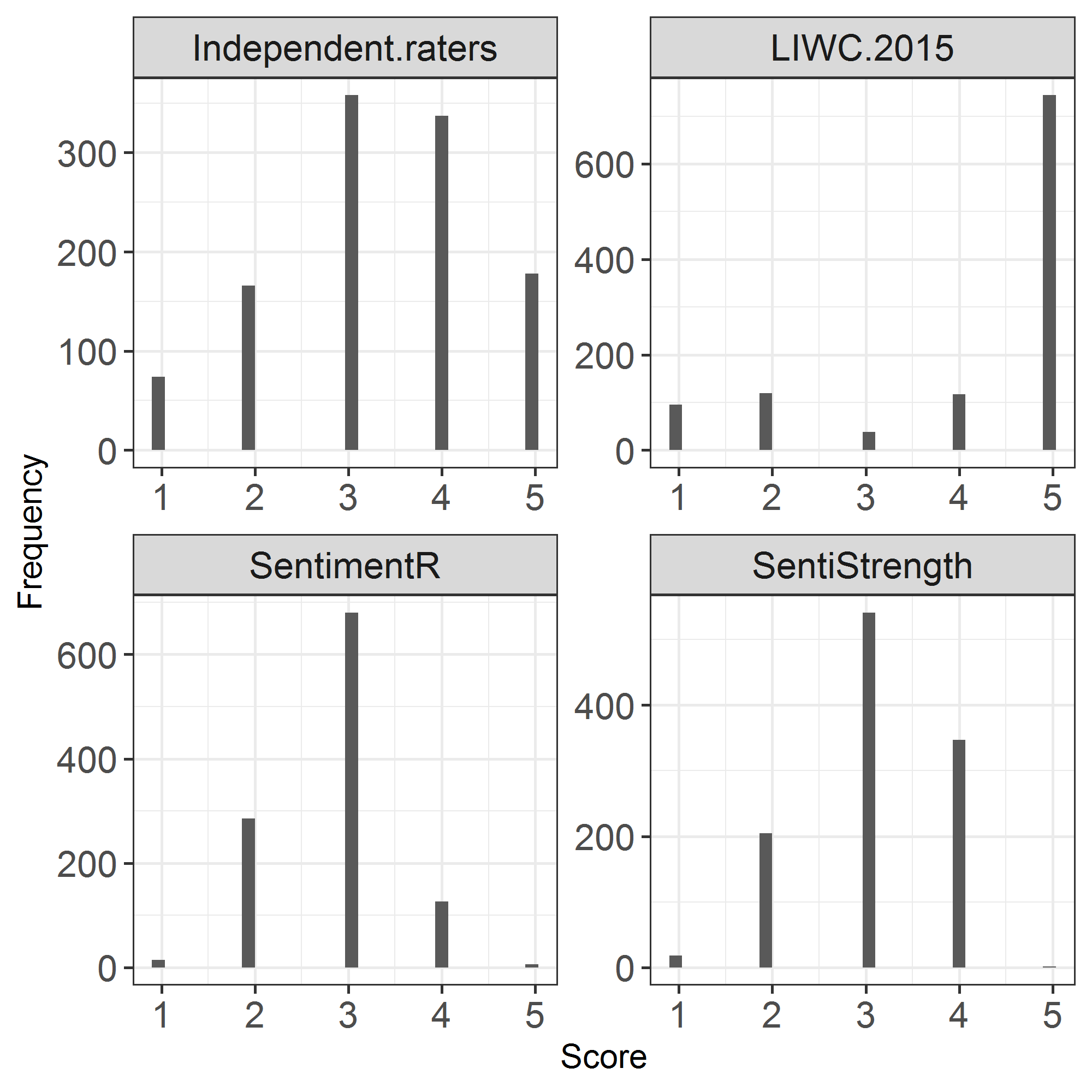

Supplement: S1 Dataset — (titled [Data_PromiseOfOpenQuestions.zip]). (ZIP) [file pone.0226408.s003.zip › Data_PromiseOfOpenQuestions/Fig1.tiff]

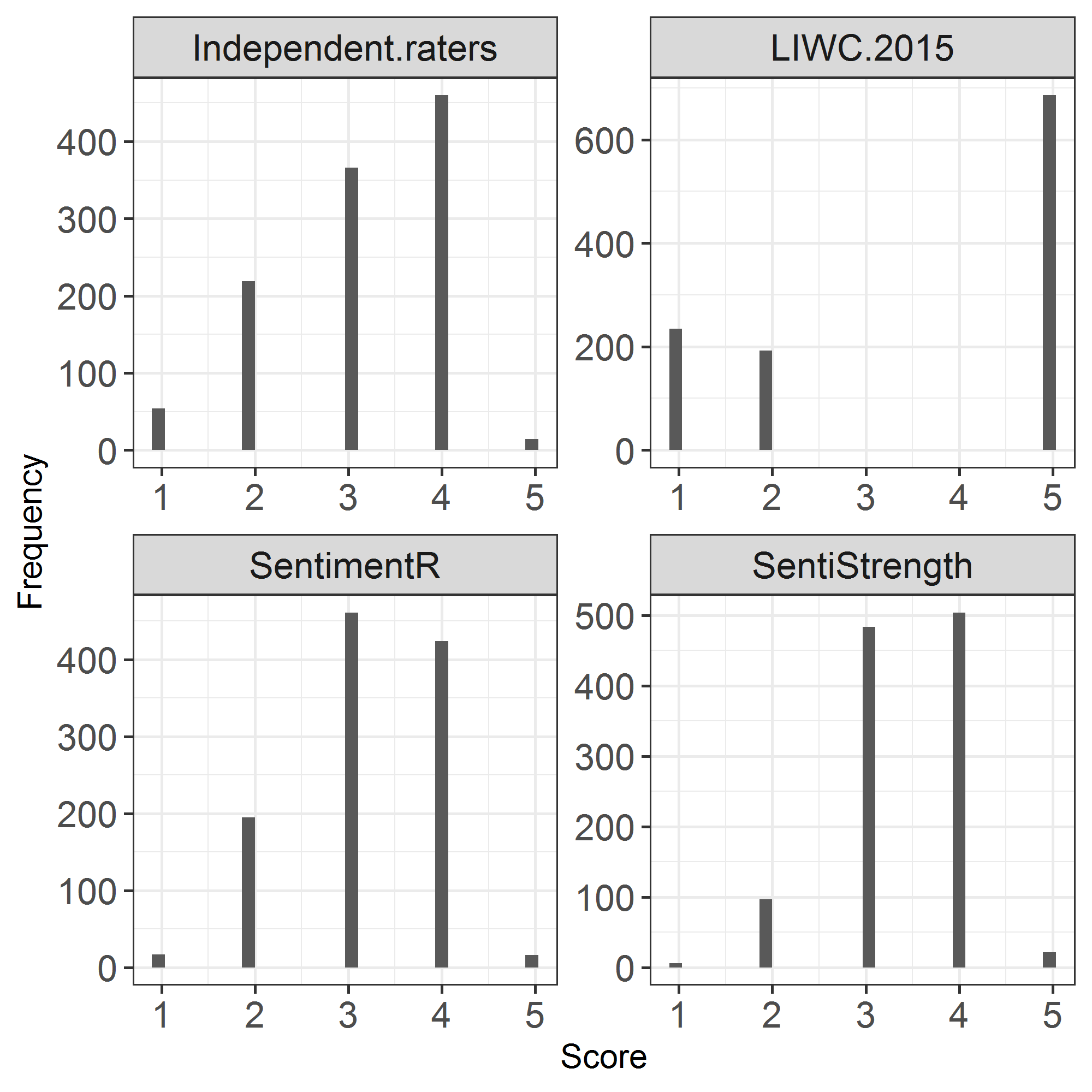

Supplement: S1 Dataset — (titled [Data_PromiseOfOpenQuestions.zip]). (ZIP) [file pone.0226408.s003.zip › Data_PromiseOfOpenQuestions/Fig2.tiff]

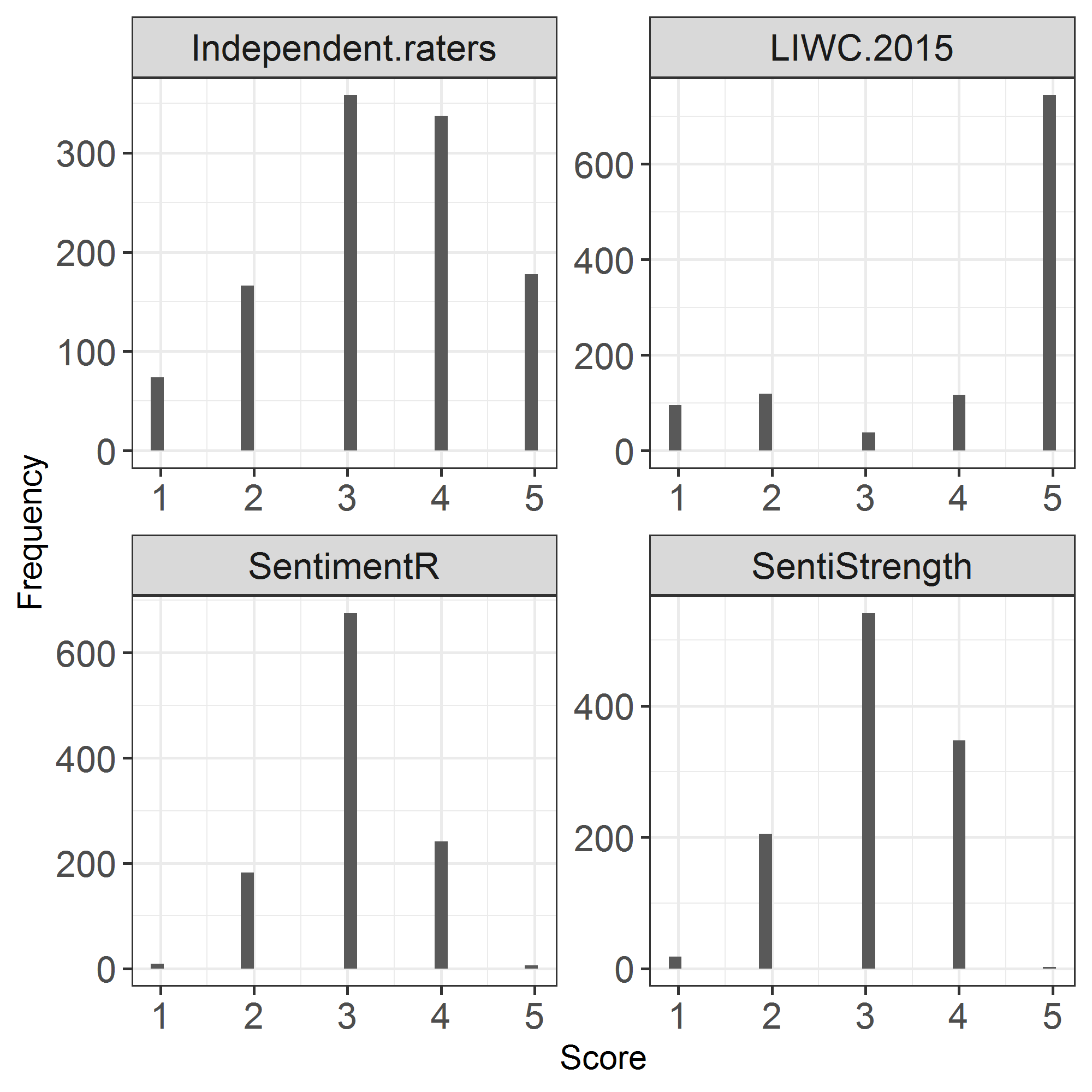

Supplement: S1 Dataset — (titled [Data_PromiseOfOpenQuestions.zip]). (ZIP) [file pone.0226408.s003.zip › Data_PromiseOfOpenQuestions/open.tiff]

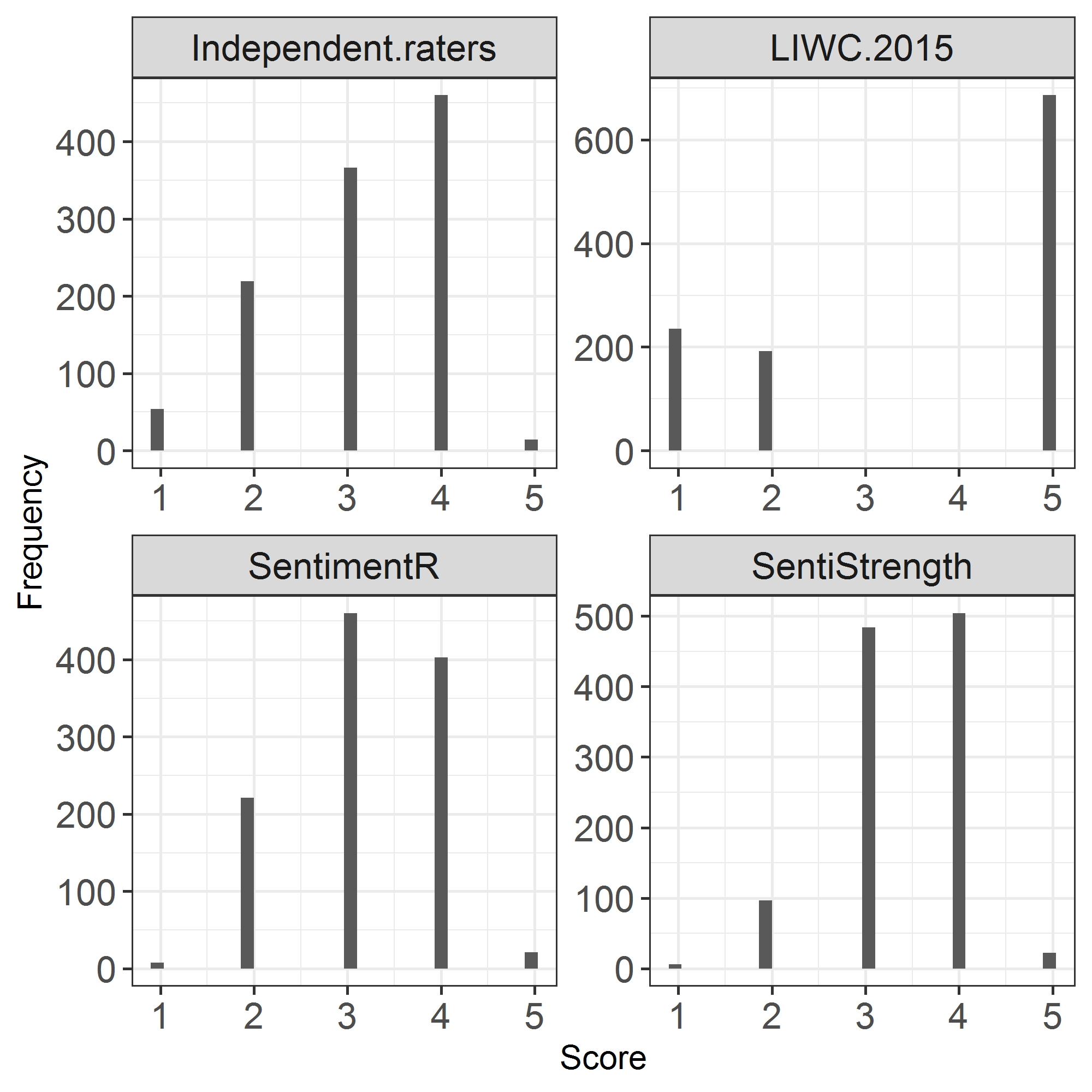

Supplement: S1 Dataset — (titled [Data_PromiseOfOpenQuestions.zip]). (ZIP) [file pone.0226408.s003.zip › Data_PromiseOfOpenQuestions/semi_open.tiff]
